# Diagnostic evaluation and clinical utility of C-MoKa for prenatal genetic testing

**DOI:** 10.1186/s12967-026-08641-w

**Published:** 2026-07-14

**Authors:** Ying Zhou, Min Xie, Liyun Tian, Juan Cao, Yuxin Zhang, Changshui Chen, Haibo Li, Jiangyang Xue

**Affiliations:** 1 The Central Laboratory of Birth Defects Prevention and Control, The Affiliated Women and Children’s Hospital of Ningbo University, 339 Liuting Street, Haishu District, Ningbo, Zhejiang 315012 China; 2 Ningbo Key Laboratory for the Prevention and Treatment of Embryogenic Diseases, The Affiliated Women and Children’s Hospital of Ningbo University, Ningbo, Zhejiang 315012 China; 3 Ningbo Key Laboratory of Genomic Medicine and Birth Defects Prevention, The Affiliated Women and Children’s Hospital of Ningbo University, Ningbo, Zhejiang 315012 China; 4 Fetal Medicine Centre, The Affiliated Women and Children’s Hospital of Ningbo University, Ningbo, Zhejiang 315012 China

**Keywords:** Prenatal diagnosis, Retrospective, Prospective, C-MoKa, Diagnostic yield, Clinical utility, Technical concordance

## Abstract

**Background:**

C-MoKa (Chromosome Conformation-based Karyotyping) is a novel 3D genome mapping platform, that enables simultaneous detection of structural variations (SVs), aneuploidies, copy number variations (CNVs), and uniparental disomy (UPD) in a single test. However, its performance in routine prenatal diagnosis is still unexplored. This study aimed to comprehensively evaluate the diagnostic performance and clinical utility of C-MoKa in prenatal diagnosis, and to assess its technical concordance with standard of care (SOC) testings in prenatal diagnosis.

**Methods:**

A two-phase study was designed. In phase 1, 56 retrospective participants with known chromosomal abnormalities (CAs) were recruited, and C-MoKa was performed on their cultured amniotic fluid (AF) samples. Concordance between C-MoKa and known CAs was analyzed. In phase 2, a prospective cohort of 208 participants for prenatal diagnosis were recruited. Samples underwent C-MoKa and karyotyping (KT), with parallel chromosomal microarray analysis (CMA) or improved whole-exome sequencing (iWES). In our study, supplementary copy number probes were enhanced in iWES detection, which could provide ~ 100 kb resolution across the genome, thus either CMA or iWES platform employed was used to assess the CNV detection. Diagnostic yields and concordance were evaluated, and the discordance of SVs and CNVs were further validated by fluorescence in situ hybridization (FISH) and CNV-seq, respectively.

**Results:**

In the retrospective cohort with known CAs, C-MoKa achieved a 94.6% (53/56) diagnostic yield and 92.8% (52/56) concordance with KT + CNV. In the prospective cohort, its diagnostic yield was 21.2% (44/208), higher than KT (12.5%, 26/208) and CNV (18.3%, 38/208). The diagnostic yield (22.6%, 47/208) was achieved when CNV platform and C-MoKa were used together. C-MoKa exhibited concordance rates of 89.4% (186/208) with KT, 89.9% (187/208) with CNV, and 90.4% (188/208) with KT + CNV. Two SVs identified by C-MoKa but missed by KT were successfully determined by FISH, and five samples with additional CNVs identified by C-MoKa were consistently detected by CNV-seq.

**Conclusions:**

Our findings demonstrate that C-MoKa is a highly effective and reliable method for prenatal diagnosis, exhibiting high concordance with SOC techniques. Compared to the conventional application of “KT + CNV” in prenatal setting, the combined use of CNV and C-MoKa appears to maximize the diagnostic yield.

**Supplementary Information:**

The online version contains supplementary material available at https://doi.org/10.1186/s12967-026-08641-w.

## Background

The global incidence of birth defects remains a significant public health challenge, with chromosomal aberrations constituting a substantial proportion of these cases [1]. The rising prevalence of birth defects, coupled with demographic shifts toward advanced maternal age, a well-established risk factor for chromosomal instability and complex anomalies, highlights the critical need for advanced prenatal diagnostic solutions [2, 3].

For decades, G-banded karyotyping (KT) has been the cornerstone of prenatal cytogenetic diagnosis for detecting aneuploidies and large structural rearrangements. However, its resolution is limited to alterations larger than 5–10 Mb, and it requires time-consuming cell culture [3, 4]. Fluorescence in situ hybridization (FISH) offers targeted, higher-resolution analysis but is not suited for genome-wide screening due to its dependence on prior knowledge for probe design [5–7]. Chromosomal microarray analysis (CMA) and low-pass whole-genome sequencing (CNV-seq) revolutionized the field by enabling genome-wide detection of copy number variants (CNVs) at a much higher resolution, but they are inherently incapable of identifying balanced chromosomal rearrangements, such as translocations and inversions, which may result in recurrent spontaneous abortions, infertility, or fetal defects [5, 8–10].

Next-generation sequencing technologies, including whole-exome sequencing (WES) and whole-genome sequencing (WGS), have further expanded the diagnostic horizon to include single-nucleotide variants (SNVs) and small insertion-deletions (indels) [11–13]. Prenatal WES has demonstrated clinical utility in fetuses with structural anomalies [12], while WGS has shown the capability to detect nearly all variant types [14, 15]. However, short-read sequencing technologies remain limited in reliably detecting balanced structural variant (SVs) and resolving complex genomic architectures [15]. Optical genome mapping (OGM) has emerged as a robust cytogenetic tool for SV detection; however, its clinical implementation faces limitations, including compromised resolution in heterochromatic regions (such as those implicated in Robertsonian translocations (RTs)), and elevated costs [16–18].

The recent integration of 3D genome architecture analysis through chromosome conformation capture techniques (e.g., Hi-C) offers a novel paradigm for genomic analysis [19–21]. The C-MoKa (Chromosome Conformation-based Karyotyping) platform harnesses this principle to deliver a comprehensive genomic profile, enabling simultaneous detection of SVs, aneuploidies, CNVs, and uniparental disomy (UPD). Its initial application in pregnancy loss analysis demonstrated significant diagnostic yield [22]. Nevertheless, its performance and integration into the standard prenatal diagnostic workflow have not been retrospectively and prospectively evaluated.

In this study, we conducted a two-phase investigation to comprehensively assess the performance of C-MoKa against current standard of care (SOC) techniques (KT, CMA, WES) in both retrospective and prospective prenatal cohorts. We aimed to determine its clinical validity, diagnostic yield, and potential utility as an effective and reliable method within the prenatal diagnostics workflow.

## Methods

### Study design

**Fig. 1:**
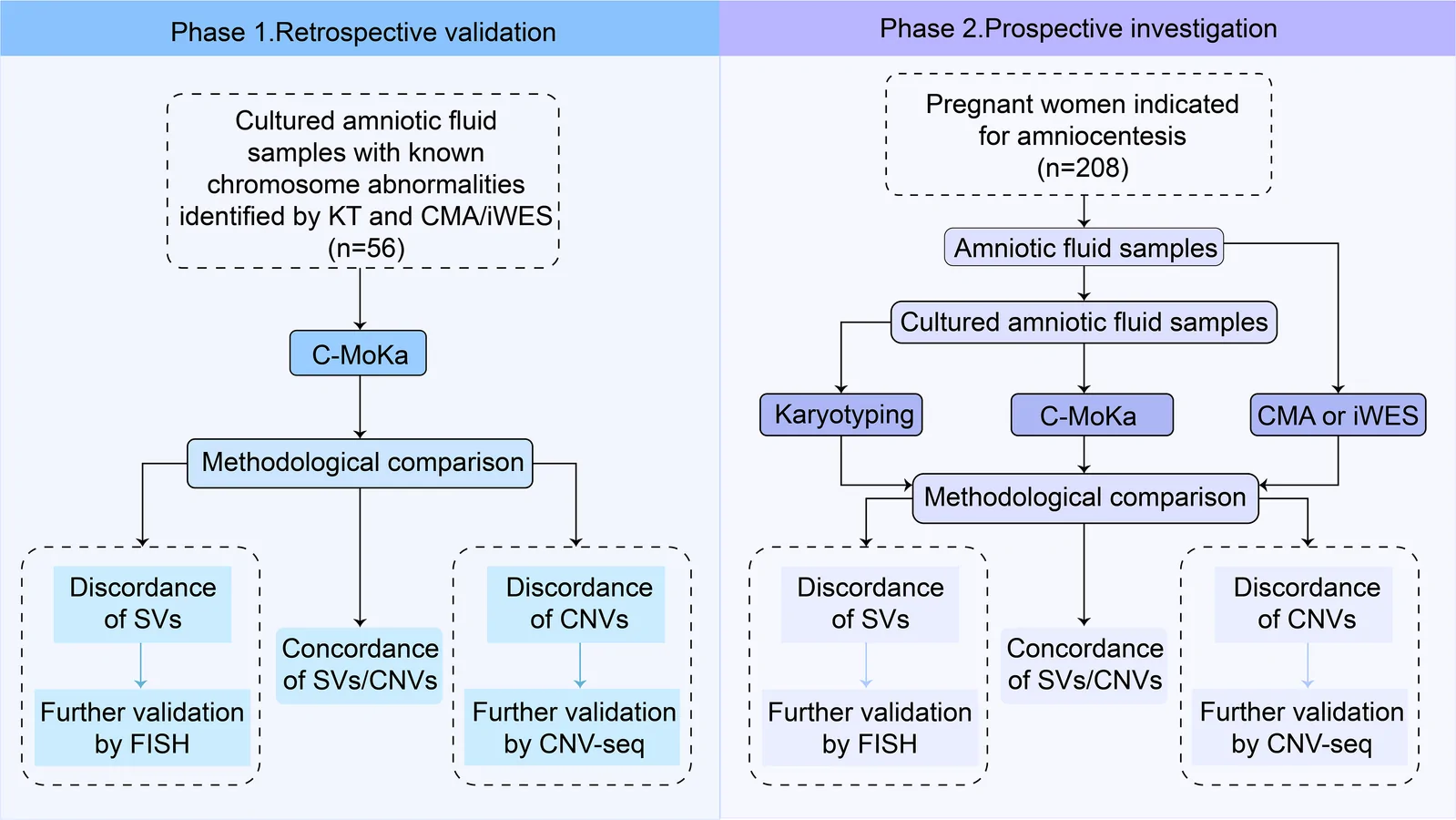
Study design. A two-phase study was conducted. In phase 1, a retrospective cohort of 56 participants with known chromosome abnormalities were recruited. Cultured amniotic fluid samples from these participants further underwent C-MoKa. The concordance of results from C-MoKa and known chromosome abnormalities was analyzed. In phase 2, a prospective group of 208 pregnant women undergoing invasive prenatal diagnosis were enlisted. Each sample was subjected to both KT and C-MoKa. Ninety-three samples underwent CMA, while the remaining 115 samples underwent WES. Diagnostic yields and technical concordance between C-MoKa and SOC testings were assessed. In both retrospective and prospective cohort, the discordance of SVs and CNVs were further validated by FISH and CNV-seq, respectively

The study design was illustrated in Fig. 1. This work was conducted in two sequential phases to comprehensively evaluate the performance of the C-MoKa platform. In our study, supplementary copy number probes were enhanced in WES detection, which could provide ~ 100 kb resolution across the genome. Prior to the present study, we validated the performance of CMA and iWES in parallel using over 1,000 prenatal clinical samples in an independent unpublished cohort, in which “clinically reportable” variants were defined as the validation benchmark. iWES platform achieved 100% diagnostic concordance for all clinically reportable CNVs and aneuploidies confirmed by CMA. For analytical purposes, both CMA and iWES were collectively designated as a unified CNV detection platform in this study.

First, a retrospective cohort of 56 cultured amniotic fluid (AF) samples with known CAs, previously identified by SOC techniques - KT, CMA, and/or WES, was recruited. KT was performed on all of 56 samples, CMA on 50 samples, and WES on 7 samples. C-MoKa analysis was subsequently carried out on all enrolled samples. We assessed the performance of C-MoKa by analyzing its concordance with the known abnormalities, focusing on its capability to detect SVs (including balanced translocation, Robertsonian translocation, unbalanced translocation, and inversion) and CNVs. Discordant results for SVs and CNVs were subjected to orthogonal validation using FISH and CNV-seq, respectively (Fig. 1).

Subsequently, a prospective cohort of 208 pregnant women undergoing invasive prenatal diagnosis between 18 and 29 weeks of gestation was enrolled to evaluate the real-world feasibility and clinical utility of C-MoKa. All samples were analyzed using both C-MoKa and KT. CMA was applied to 93 samples, and WES was used for the remaining 115 samples. Again, CMA and WES were collectively considered as the CNV platform. The diagnostic yields and technical concordance among C-MoKa, KT, and the CNV platform were systematically compared. Any discordant SV or CNV findings were validated by FISH and CNV-seq, respectively (Fig. 1). Notably, limited residual cryopreserved samples and ambiguous breakpoints of partial complex SVs led to failed probe design and validation for several discordant cases.

**Fig. 2:**
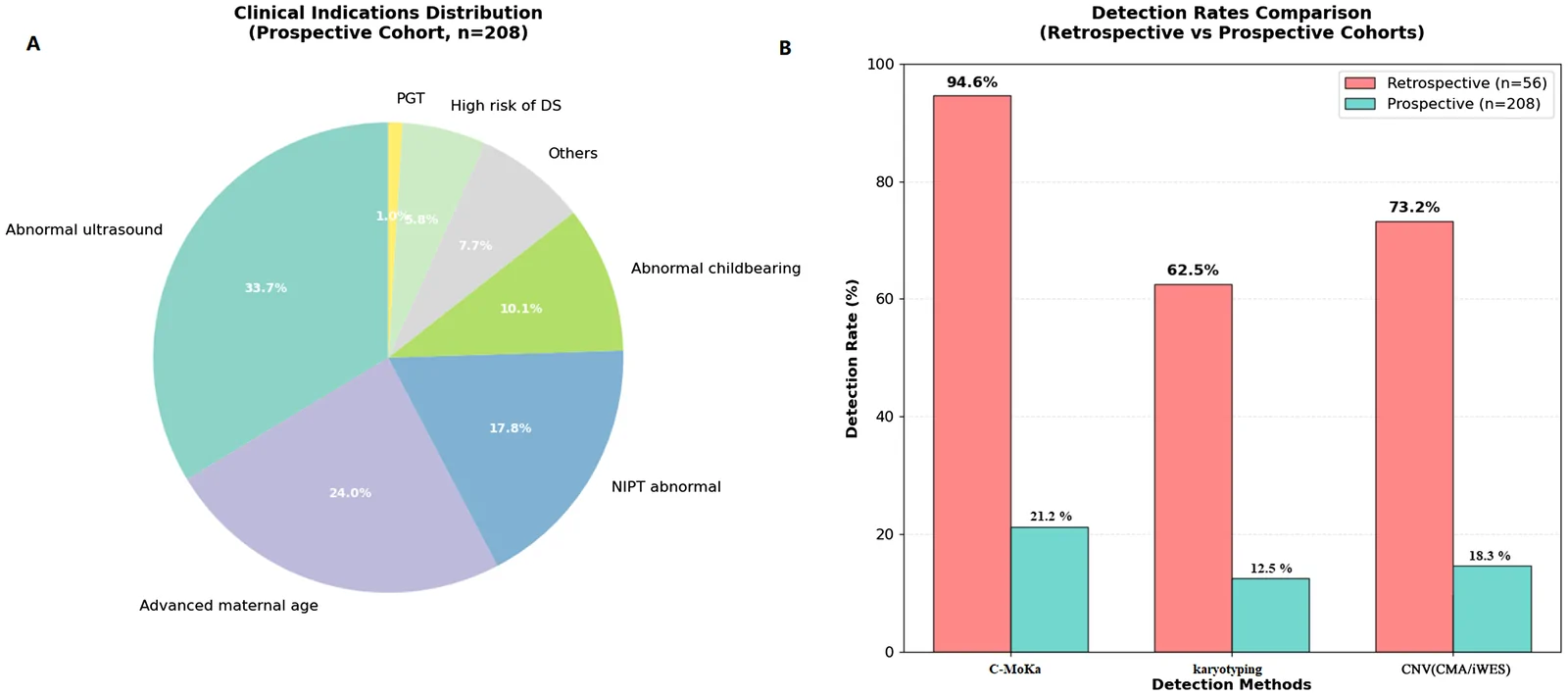
Clinical characteristics and method performance. Clinical indications distribution in the prospective cohort (**A**) and Comparison of detection rates between retrospective and prospective cohorts (**B**). The prospective cohort (*n* = 208) showed diverse clinical indications with abnormal ultrasound being the most common (33.7%). Detection rates were significantly higher in the retrospective cohort with known abnormalities compared to the prospective cohort representing real-world clinical scenarios

The distribution of clinical indications in this prospective cohort and the comparison of detection rates in both cohorts were detailed in Fig. 2A and B, respectively. Written informed consent was obtained from all participants, and the study was approved by the Institutional Review Board (IRB) of the Affiliated Women and Children’s Hospital of Ningbo University (No. EC2023-094). The study complied with the principles of the Declaration of Helsinki as revised in 2013.

### Cytogenetic and genomic analyses of SOC testings

#### G-banded karyotyping

Following amniocentesis, amniotic fluid cells were cultured, harvested, and prepared for slide-making using the conventional cell culture flask method, as established by the Comprehensive Birth Defect Prevention Laboratory at our institution. Karyotype analysis was conducted using G-banding. A total of 25 metaphase spreads were counted, and five karyotypes were analyzed in detail. Chromosomal karyotypes were reported in accordance with the International System for Human Cytogenomic Nomenclature (ISCN 2020).

#### Chromosomal microarray analysis

For CMA, genomic DNA was extracted from fresh amniotic fluid and hybridized onto the Affymetrix Cytoscan 750 K Array chip following the manufacturer’s instructions. CNV data were analyzed with reference to public databases (e.g., DGV, DECIPHER, OMIM, and UCSC) and classified according to the guidelines of the American College of Medical Genetics and Genomics (ACMG).

#### Improved whole-exome sequencing

Improved WES (iWES) was performed with a custom exome capture kit. Firstly, genomic DNA from fresh amniotic fluid was sequenced on the MGIDNBSEQ-T7 platform (MGI Tech Co., Ltd., Shenzhen). Targeted region capture was performed using hybridization capture reagents (Twist Bioscience, USA), covering exons of approximately 20,000 genes along with supplementary enhanced copy number probes that provided ~ 100 kb resolution across the genome. Bioinformatic analysis was conducted as previously described. Variants and CNVs were interpreted for pathogenicity based on ACMG guidelines [23, 24].

### Prenatal C-MoKa workflow

#### Amniotic fluid sample processing

Cultured amniotic fluid samples were fixed with 2% formaldehyde (FA) for 10 min at room temperature. The fixation reaction was quenched with glycine (final concentration 128 mM) for at least 15 min at room temperature and on ice. Cells were washed with PBS, pelleted, and flash-frozen in aliquots using liquid nitrogen for subsequent analysis.

#### C-MoKa protocol and sequencing

**Fig. 3:**
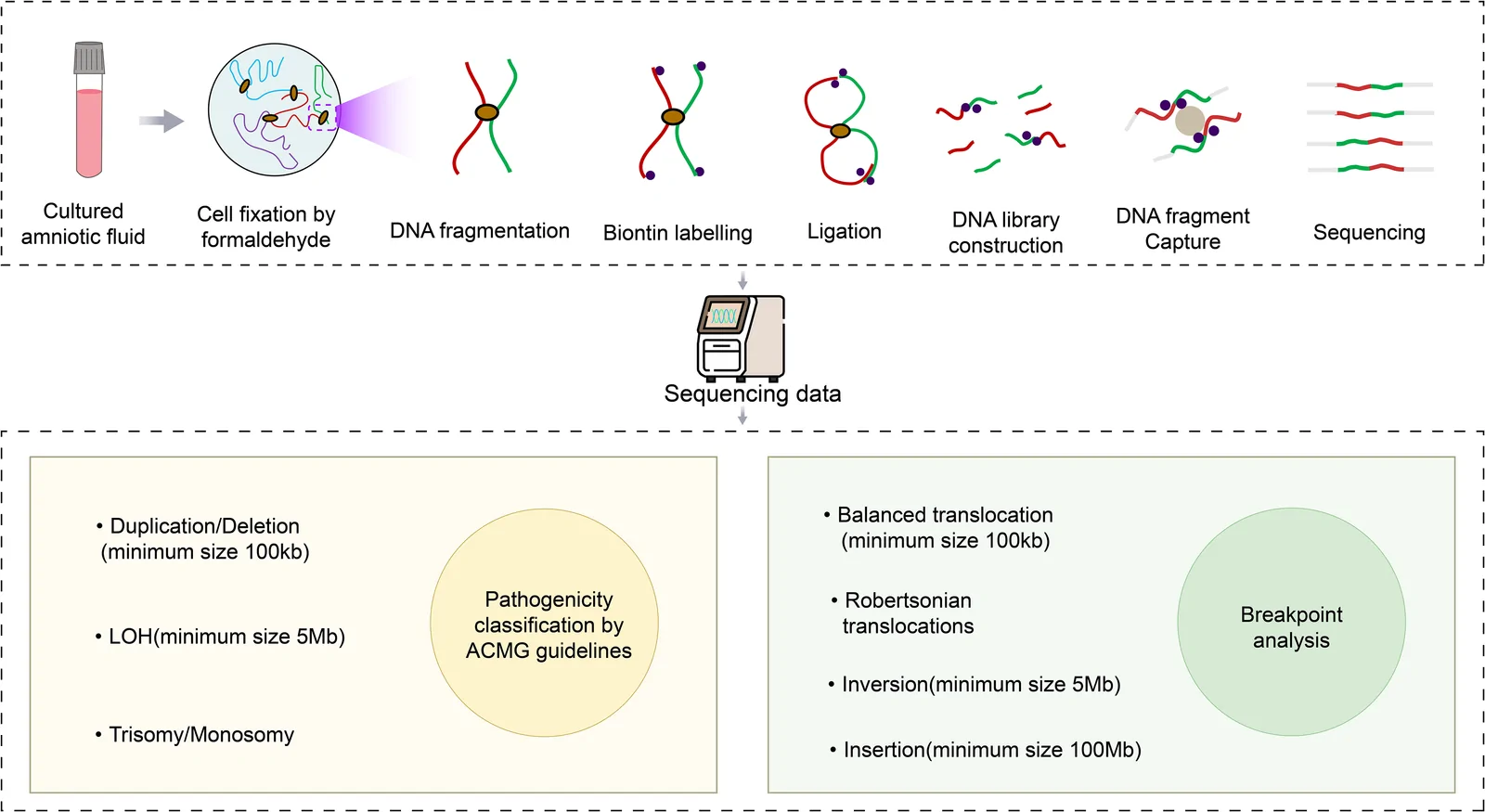
Work flow of C-MoKa. During wet lab process, cultured amniotic fluid samples were collected and fixed by formaldehyde (FA). After fixed cells were lysed, digested, labeled and ligated, DNA library was prepared and captured, and subsequently sequenced. Then, SVs (including balanced translocation, Robertsonian translocations, inversion and insertion), CNVs (including duplication/deletion, trisomy/monosomy), and LOH/UPDs were investigated

Chromosome conformation capture was performed as previously described and outlined in Fig. 3. Briefly, FA fixed cells were lysed, digested, labeled with biotin-11-dATP (AAT Bioquest, 17014) and ligated. After crosslink reversal and purification, a DNA library was constructed using a commercial library preparation kit (Cat. No KT100804048, Yikon Genomics Ltd). Biotin-labeled DNA fragments were captured using magnetic beads (Invitrogen, 65001) and sequenced on the BGI DNBSEQ-T7 platform with 30 million read-pairs.

#### SV and CNV analysis

The core principles and analytical workflow for C-MoKa SV/CNV variant calling adopted in this study were consistent with those reported by Bao X et al. [22]. Briefly, the overall procedure encompassed reference matrix construction, sliding-window CNV detection, and integrative analysis of spatial contact intensity and copy number data. Raw sequencing data were processed using Juicer v1.6 to generate an initial spatial contact matrix. A reference system was constructed using 22 negative control samples without SVs. A normalized spatial contact matrix was generated by correcting with this reference system, and spatial contact intensity was assessed within a 1 Mb window. Putative SVs were identified when the observed/expected contact ratio ≥ 2. To enhance accuracy and reduce false positives in CNV calling, we integrated CNV sequencing data with the spatial contact matrix. First, copy numbers for genomic regions were determined using a sliding window algorithm; regions with uniform copy number deviations from the expected diploid state (2 copies) were considered candidate CNVs. Second, candidate regions are flagged when contact intensity < 0.7 (deletion) or > 1.3 (duplication), and final calls require overlap with copy-number deviations from the diploid state.

With regard to false-positive filtering and handling of repetitive/pericentromeric regions, the proprietary pipeline employs a multi-step filtering strategy that includes removal of spurious contacts supported by fewer than three read pairs, exclusion of regions with low mappability scores (based on the 1kGP mappability track), and a requirement that candidate breakpoints are supported by at least five uniquely mapped read pairs. For pericentromeric and telomeric satellite repeats, which exhibit intrinsically low mappability, the pipeline does not make primary calls in these regions; any potential variant identified there is flagged for orthogonal validation.

To benchmark our pipeline’s performance against an independent standard, we additionally tested C-MoKa on a panel of 13 well-characterized cell lines (9 with known structural abnormalities confirmed by G-banded karyotyping, including 8 balanced translocations and 1 Robertsonian translocation; and 4 negative controls). C-MoKa achieved 100% concordance with the karyotyping results in this validation set, with no false positives or negatives.

For mosaic aneuploidies and CNVs, the reliable detection threshold was set at 20% under our current analytical pipeline and sequencing depth (~ 30 million read-pairs). Variants with mosaic ratio below 20% are prone to missed detection.

#### LOH/UPD analysis

For loss of heterozygosity (LOH) and UPD analysis, the LD-PGTA software was employed. This tool uses known population haplotypes from the 1000 Genomes Project (1kGP) to infer haplotype combinations in each genomic region. For disomic and monosomic regions, the likelihood ratio of haplotypes was calculated. A 1:1 ratio indicated a normal heterozygous segment, whereas a 1:0 ratio suggested LOH or UPD, reflecting the presence of only one haplotype.

### Additional practical information of C-MoKa

The complete C-MoKa workflow from sample receipt to clinical report is outlined below: 7 days of cell culture, 0.5 days of manual formaldehyde fixation, overnight automated library preparation, 1–2 days of paired-end sequencing on DNBSEQ-T7 (30 million read pairs per sample), plus 1–2 days of variant calling and report interpretation for SVs, CNVs and LOH. The full turnaround time totals 10–11 days. Owing to the continuous decline in next-generation sequencing costs, we have set the total price of the C-MoKa test at ¥2,500 RMB (approximately US$350) per sample. The comparison of turnaround times (TAT) and total costs between C-MoKa and SOC testing (CMA, iWES, and KT) is provided in the supplementary materials.

### FISH and CNV-seq validation

For FISH validation, frozen cultured amniotic fluid samples were used. DNA fusion probes were designed based on breakpoints identified by C-MoKa. After hybridization and dehydration, slides were air-dried and mounted in FluorShine containing Hoechst 33,342 (Yikon, FluorShineTM). Images were acquired using a Leica DM2000 fluorescence microscope with a 100×/1.40 NA oil-immersion objective.

For CNV-seq, DNA fragment libraries were constructed from 50 ng of DNA per sample and sequenced on the MGISEQ-500 platform (single-end 35 bp). Standard bioinformatic analysis was performed after generating FASTQ data.

## Results

### Concordance between C-MoKa and SOC testings in the retrospective cohort

To validate the performance of C-MoKa in detecting chromosomal aberrations, we first analyzed a retrospective cohort of 56 clinical samples with known CAs (Supplementary Table 1). C-MoKa was applied to cultured amniotic fluid samples from these participants. Following sequencing data analysis and pathogenicity evaluation, C-MoKa achieved an overall diagnostic yield of 94.6% (53/56) (Table 1 & Supplementary Table 1). Regarding SVs, C-MoKa demonstrated 100% concordance with KT, successfully identifying all 12 previously known SV cases. C-MoKa detected 12 instances of aneuploidy (monosomies or trisomies), 11 of which had been previously identified; the remaining one was a case of mosaic monosomy X (patient A241079) previously detected by KT with a high mosaicism rate of 80%. Among seven other previously identified mosaic aneuploidies, C-MoKa consistently detected four, while it failed to detect the remaining three, which exhibited relatively low mosaicism rates (patient A240661: 20.3% by KT; A240904: 11.8% by KT; A241237: 14.8% by KT). Also, C-MoKa identified all 23 pre-characterized CNVs in 23 cultured AF samples; additionally, it discovered a novel duplication in patient A240965. Consequently, the overall concordance rate between C-MoKa and the combined results of KT, CNV (CMA/iWES) was 92.8% (52/56).

**Table 1:** The diagnostic yield and concordance of testing results by different testing strategies in the retrospective cohort

Number of cases with testing results identified by different testing strategies								Concordance rate		
Type of variants	KT	CNV^$^	C-MoKa	KT+ CNV^$^	KT+ C-MoKa	CNV^$^+ C-MoKa	KT + CNV^$^+ C-MoKa	C-MoKa to KT (%)	C-MoKa to CNV^$^ (%)	C-MoKa to KT + CNV^$^ (%)
Balanced Translocation	6	0	6	6	6	6	6	100.0 (6/6)	/	100.0 (6/6)
Robertsonian translocation	4	0	4	4	4	4	4	100.0 (4/4)	/	100.0 (4/4)
Unbalanced Translocation	1	0	1	1	1	1	1	100.0 (1/1)	/	100.0 (1/1)
Inversion	1	0	1	1	1	1	1	100.0 (1/1)	/	100.0 (1/1)
Monosomy/Trisomy	12	12	13	12	13	13	13	92.3 (12/13)	92.3 (12/13)	92.3 (12/13)
Mosaic Monosomy/Triosomy	8	6	4	8	7	5	7	50.0 (4/8)	75.0 (4/6)	50.0 (4/8)
Deletion/Duplication	1	23	24	23	24	24	24	4.2 (1/24)	95.8 (23/24)	95.8 (23/24)
Mosaic Deletion/Duplication	1	0	1	1	2	1	2	0.0 (0/1)	0.0 (0/1)	0.0 (0/1)
ROH	0	2	2	2	2	2	2	0.0 (0/2)	100.0 (2/2)	100.0 (2/2)
psu idic	1	0	0	1	1	0	1	0.0 (0/1)	/	0.0 (0/1)
Total number of positive cases	35	43	53	56	56	54	56	56.6 (30/53)^*^	97.7 (42/43)^#^	98.1 (52/53)^$^
Total number of cases without clinical significant variant	21	13	3	0	0	2	0	0.0 (0/3)	66.7 (2/3)	0.0 (0/3)
Diagnostic yield (%)	62.5	74.1	94.6	100.0	100.0	96.4	100.0	/	/	/
Overall concordance rate	/	/	/	/	/	/	/	53.6 (30/56)	95.7 (44/46)	92.8 (52/56)

Note:^$^, indicated chromosome abnormalities detected by CMA and iWES

^*^, included an extra case with mosaic monosomy-X (80%) detected by Karyotyping, and with monosomy-X detected by C-MoKa, those results of case were considered consistent

^#^, includedd an extra case with mosaic monosomy-X (66%) detected by CMA, and with monosomy-X detected by C-MoKa, those results of case were considered consistent

^$^, included an extra case with mosaic monosomy-X (66%) detected by CMA, mosaic monosomy-X (80%) detected by Karyotyping, and with monosomy-X detected by C-MoKa, those results of case were considered consistent

### Diagnostic yield of C-MoKa, KT, and CNV detection in the prospective cohort

**Fig. 4:**
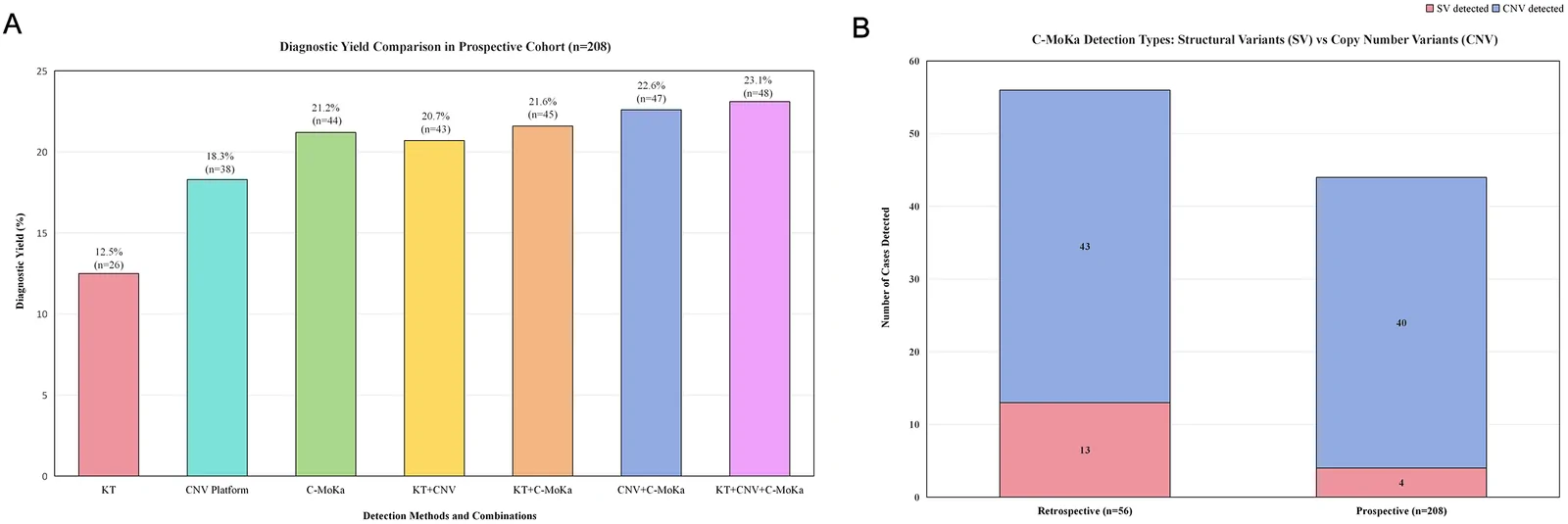
C-MoKa diagnostic yield and detection capabilities analysis. Comprehensive diagnostic yield comparison among different methods and combinations in prospective cohort (**A**) and Detailed analysis of C-MoKa detection types (**B**). Compared to the conventional implement of “KT + CNV” combination in prenatal diagnosis, “CNV + C-MoKa” yielded more diagnostic outcomes of 22.6% (47/208) in the prospective cohort. C-MoKa demonstrated superior CNV detection in both cohorts

We next evaluated the feasibility and clinical utility of C-MoKa in a prospective cohort of 208 participants (Supplementary Table 2). As summarized in Table 2; Fig. 4A, the diagnostic yield was 12.5% (26/208) for KT, 18.3% (38/208) for the CNV platform, and 21.2% (44/208) for C-MoKa. Upon integrating the outputs from both technologies, the CNV + C-MoKa combination achieved the highest diagnostic yield of 22.6% (47/208), compared to 21.6% (45/208) for KT + C-MoKa and 20.7% (43/208) for KT + CNV (Table 2; Fig. 4A). The combination of all three platforms (KT + CNV+C-MoKa) yielded a diagnostic rate of 23.1% (48/208), marginally higher than that of CNV + C-MoKa. The SV detection yield was identical for both KT and C-MoKa at 1.9% (4/208), although the specific sets of four participants identified by each method were distinct.

**Table 2:** The diagnostic yield and concordance of testing results by different testing strategies in the prospective cohort

Number of cases with testing results identified by different testing strategies								Concordance rate		
Type of variants	KT	CNV^$^	C-MoKa	KT+ CNV^$^	KT+ C-MoKa	CNV^$^+ C-MoKa	KT + CNV^$^+ C-MoKa	C-MoKa to KT (%)	C-MoKa to CNV^$^ (%)	C-MoKa to KT + CNV^$^ (%)
Balanced Translocation	3	0	3	3	4	3	4	66.7 (2/3)	/	66.7 (2/3)
Inversion	1	0	2	1	2	2	2	0.0 (0/2)	/	0.0 (0/2)
Monosomy/Triosomy	15	15	15	15	15	15	15	100.0 (15/15)	100.0 (15/15)	100.0 (15/15)
Mosaic Monosomy/Trisomy	3	2	5	3	5	5	5	60.0 (3/5)	40.0 (2/5)	60.0 (3/5)
Deletion/Duplication	3	19	17	19	17	20	20	17.6 (3/17)	94.1 (16/17)	88.2 (15/17)
Mosaic Deletion/Duplication	0	0	2	0	2	2	2	0.0 (0/2)	0.0 (0/2)	0.0 (0/2)
ROH	0	2	1	2	1	2	2	0.0 (0/2)	50.0 (1/2)	50.0 (1/2)
psu idic	1	0	0	1	1	0	1	0.0 (0/1)	/	0.0 (0/1)
Total number of positive cases	26	38	44	43	45	47	48	52.3 (23/44)	85.0 (34/40)	81.8 (36/44)
Total number of cases without clinical significant variant	182	170	164	165	163	161	160	99.4 (163/164)	93.3 (153/164)	92.7 (152/164)
Diagnostic yield (%)	12.5	18.3	21.2	20.7	21.6	22.6	23.1	/	/	/
Overall concordance rate	/	/	/	/	/	/	/	89.4 (186/208)	89.9 (187/208)	90.4 (188/208)

Note:^$^, indicated chromosome abnormalities detected by CMA and iWES

### Concordance between C-MoKa and SOC testings in the prospective cohort

**Fig. 5:**
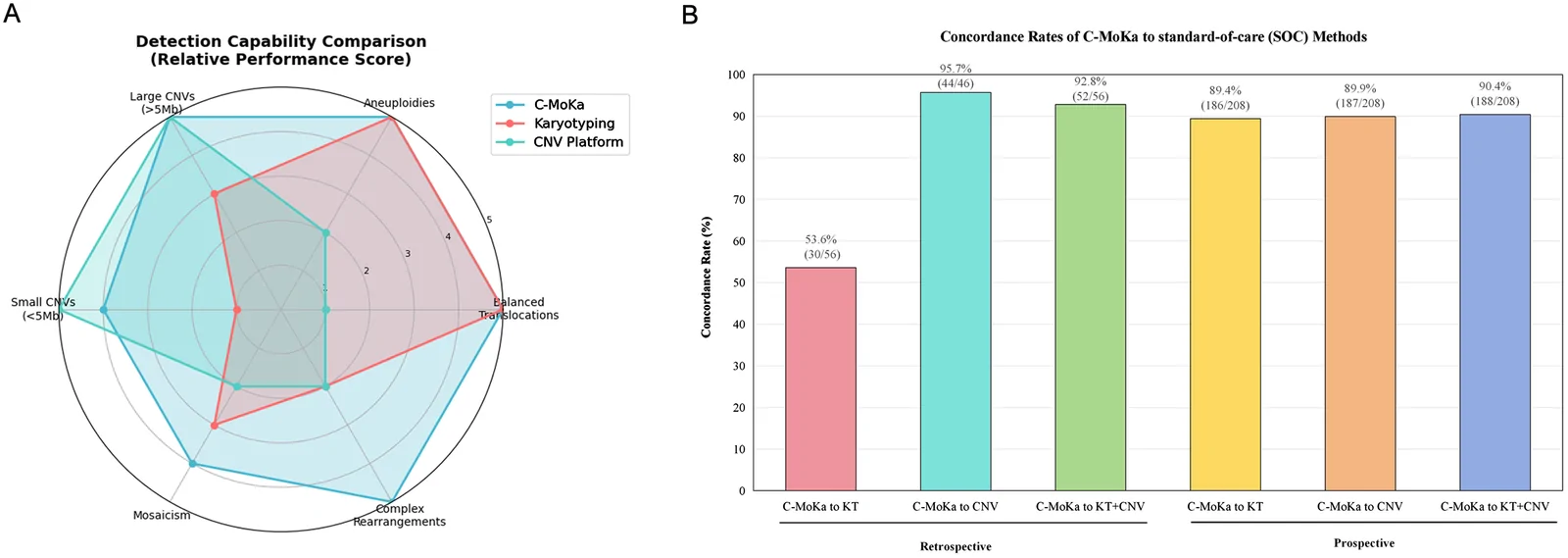
Comparative detection capabilities and concordance analysis. Radar chart comparing detection capabilities of C-MoKa across different variant types (**A**) and Concordance rates between C-MoKa and SOC methods in both retrospective and prospective cohorts (**B**). C-MoKa demonstrated comprehensive detection capabilities across multiple chromosomal abnormality types and showed high concordance (89.4–92.8%) with established methods

MoKa alone identified 44 cases with pathogenic CAs (40 CNVs & 4 SVs) in the prospective cohort (Fig. 4B). The spectrum of abnormalities detected included: four cases with SVs (9.1%), 15 cases with non-mosaic aneuploidies (34.1%), five cases with mosaic aneuploidies (11.4%), 17 cases with deletions/duplications (38.5%), two cases with mosaic deletions/duplications (4.6%), and one case with runs of homozygosity (ROH) (2.3%) (Table 2). Compared with prenatal SOC testings, C-MoKa platform demonstrated more comprehensive detection capabilities across multiple chromosomal abnormality types (Fig. 5A).

C-MoKa demonstrated 52.3% (23/44) concordance with KT, while KT failed to detect 19 positive cases identified by C-MoKa, with two additional cases showing discordant results between platforms. Among the four SVs identified by C-MoKa, two balanced translocations were also consistently detected by KT. However, C-MoKa identified an inversion in sample A241520 and a complex SV in sample A241632, whereas KT reported a normal karyotype for A241520 and only a suspected inversion for A241632. Conversely, a suspected mosaic balanced translocation involving three chromosomes in sample A241672 identified by KT was not detected by C-MoKa. C-MoKa demonstrated 85.0% concordance (34/40) with the CNV platform, with the CNV platform missing nine positive cases detected by C-MoKa and one case showing discordant results. The concordance rate between C-MoKa and the combined KT + CNV results was 81.8% (36/44), with KT + CNV missing five positive cases identified by C-MoKa and three cases yielding discordant findings. The concordance rates between different methods are presented in Fig. 5B.

### Diagnostic performance metrics of C-MoKa for different variant types

To further characterize the diagnostic accuracy of C-MoKa, we calculated sensitivity, specificity, positive predictive value (PPV), and negative predictive value (NPV) with 95% confidence intervals (CIs) for C-MoKa across different variant types (SVs, CNVs, aneuploidies, mosaicism, and ROH) within our prospective cohort (*n* = 208). The combined results of KT and CNV platforms were used as the reference standard for these calculations. These data are presented in Supplementary Table 4. Our analysis presented an overall sensitivity of 88.6%, specificity of 99.5%, PPV of 88.6%, and NPV of 99.5% for C-MoKa across all variant types. Notably, C-MoKa achieved perfect diagnostic efficacy, with 100% sensitivity, specificity, PPV and NPV for SVs and aneuploidies. Regarding CNVs, C-MoKa yielded a sensitivity of 84.2%, specificity of 99.5%, PPV of 94.1%, and NPV of 98.4%. However, lower sensitivities were observed for mosaicism (75.0%) and ROH (50.0%), indicating the technical limitations of C-MoKa in detecting low-level mosaic events and ROH.

### Validation of discordant SVs by FISH

**Fig. 6:**
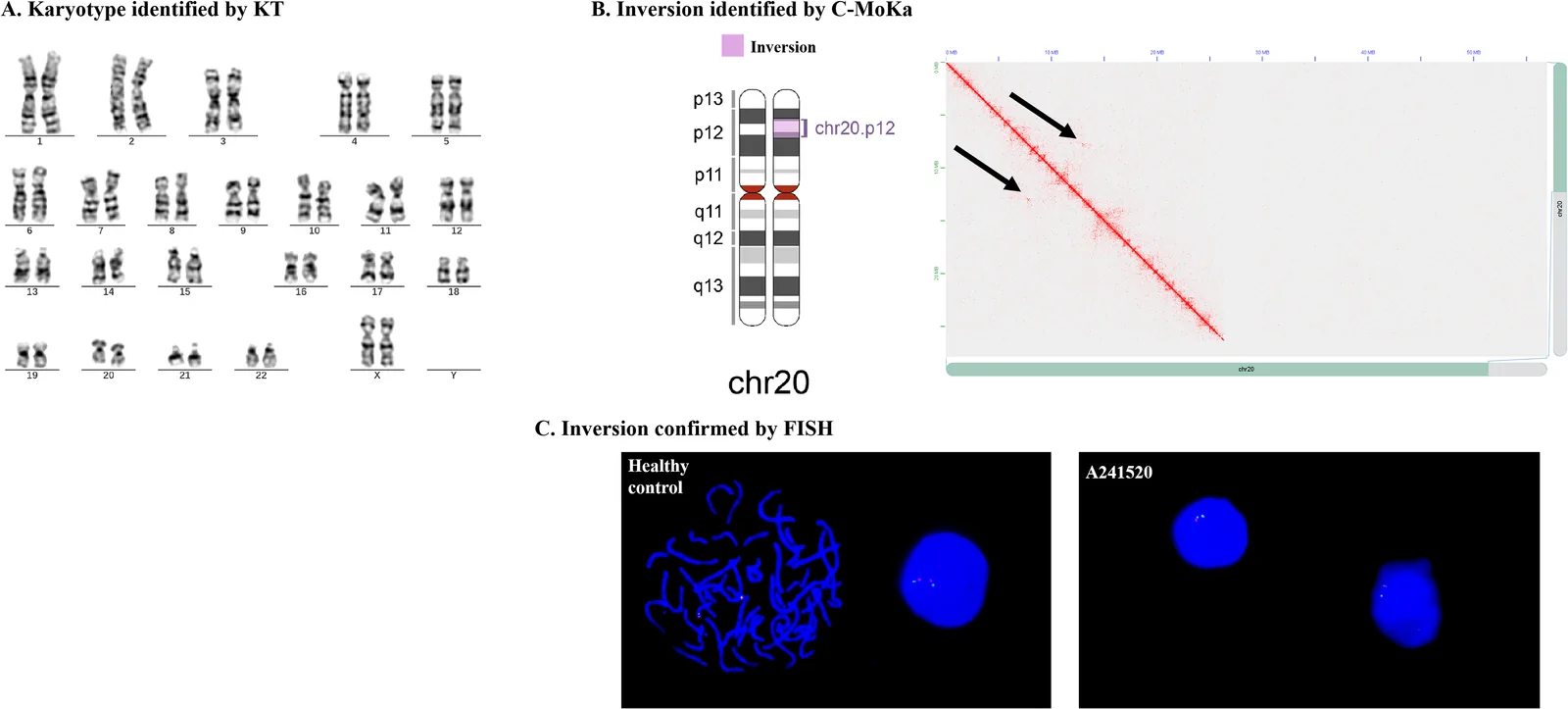
KT, C-MoKa and FISH results in A241520. (**A**) KT showed normal karyotype in A241520; (**B**) C‑MoKa revealed an inversion, 46,XX, inv(20)(p12.3p12.1). The black arrows indicate the two inversion breakpoints, demonstrating that the off‑diagonal signal shift in the contact matrix is mirror‑symmetric relative to the normal diagonal; (**C**) FISH confirmed the inversion identified by C-MoKa

Across both cohorts, five samples with discordant SV calls between C-MoKa and KT + CNV were identified and subjected to validation by FISH (Supplementary Table 3). However, reliable FISH validation could not be achieved for three SVs due to both the absence of precisely mapped breakpoints and insufficient cryopreserved cultured amniocytes. For the two additional SVs detected by C-MoKa with precisely mapped genomic coordinates, breakpoint-spanning fusion probes were successfully designed, and subsequent FISH analysis confirmed these rearrangements. A representative case A241520, where C-MoKa identified an inversion, 46,XX, inv(20)(p12.3p12.1), while KT showed a normal karyotype. Fusion probes spanning the breakpoints at 20p12.3 and 20p12.1 were used. As shown in Fig. 6, FISH confirmation of the inversion was demonstrated by either yellow fusion signals or closely adjacent red/green signals, indicating recombination between the distal 20p12.3 fragment and the 20p12.1 region.

### Validation of discordant CNVs by CNV-seq

Across both cohorts, 10 samples with discordant CNV calls (excluding mosaic aneuploidies with extremely low mosaicism rates detected by KT) were identified and further validated by CNV-seq (Supplementary Table 3). CNV-seq data from four samples were of poor quality and excluded due to insufficient sample availability. Among the remaining six samples, CNV-seq results for four cases were consistent with the CNVs identified by C-MoKa. The corresponding CNVs in the other two cases, which had low mosaicism ratios, were not detected by CNV-seq. The successfully validated discordant results partially support the detection accuracy of C-MoKa. A portion of discordant cases could not be fully validated due to limited sample resources, and further verification with larger sample cohorts is required.

## Discussion

Congenital anomalies, defined by the World Health Organization as structural or functional abnormalities occurring during intrauterine life, represent a significant global health burden, affecting an estimated 3–6% of births [25, 26]. In this context, prenatal diagnosis is paramount for the early detection of genetic alterations underlying these defects. The current clinical arsenal includes various technologies, each with inherent strengths and limitations. KT has long been the cytogenetic cornerstone, capable of detecting aneuploidies and large chromosomal rearrangements, but its clinical utility is constrained by a limited resolution of 5–10 Mb [27]. In recent years, CMA is recommended as a first-line test for rapid detection of aneuploidies and microdeletions/duplications, offering ~ 6% incremental yield over KT [8]. However, CMA cannot detect balanced structural variations ( e.g. balanced translocations and inversions), which variations are important genetic factors leading to recurrent miscarriages, infertility, and some congenital malformations [28]. Prenatal exome sequencing has been increasingly used when fetal structural anomalies are detected on ultrasound examination, which can detect “pathogenic variants” to the resolution of a single base pair, in addition to aneuploidies and CNVs [29]. Both technologies exhibit suboptimal performance in detecting SVs.

The C-MoKa technology is founded on three-dimensional genome conformation capture. It integrates high-throughput sequencing with bioinformatics algorithms to reconstruct the whole-genome chromatin spatial interaction map, thus enabling high-resolution identification of chromosomal SVs [30]. Previous research has shown that C-MoKa can not only precisely locate the breakpoints of various SVs, including translocations, inversions, and insertions (with a resolution of up to 100 kb), but also detect CNVs and UPD simultaneously. It demonstrates superior diagnostic efficacy compared to traditional KT in screening the genetic causes of recurrent miscarriage and infertility [22, 31]. To date, there have been no reports on the application of C-MoKa in prenatal diagnosis. This study is the first to explore the diagnostic yield and clinical utility of C-MoKa technology in prenatal diagnosis. A real-world cohort was established through a combination of retrospective and prospective study designs.

Our data demonstrated the potential utility of C-MoKa as an effective and reliable method within the prenatal diagnostics workflow, which achieved an overall sensitivity of 88.6%, specificity of 99.5%, PPV of 88.6%, and NPV of 99.5% across different variant types. Notably, C-MoKa displayed perfect diagnostic efficacy in detection of SVs and aneuploidies, but lower sensitivities in mosaicism detection. The high overall NPV of 99.5% indicated that negative C-MoKa results can substantially reduce the likelihood of pathogenic chromosomal abnormalities, supporting its utility as a dependable tool for routine prenatal screening.

This study achieved a diagnostic yield of 21.2% in a prospective cohort, outperforming KT (12.5%) and CNV platform (18.3%). Critically, combining two technologies, the integration of C-MoKa with CNV detection yielded the highest detection rate (22.6%), underscoring its complementary role. The high concordance with established techniques was 89.4–90.4%. The successfully verified discordant results support the good detection accuracy of C-MoKa; for the unverified cases due to sample limitation, further sample accumulation and multi-center verification are still needed. Furthermore, the ability of C-MoKa to detect balanced SVs, which are often missed by CNV-based methods, highlights its unique advantage in providing a more comprehensive genomic assessment [32].

Consistent with prior reports of an approximate 10% diagnostic yield for KT in prenatal diagnosis [8], the prospective cohort in the present study achieved a diagnostic yield of 12.5% (26/208) by KT, therein four cases carried with SVs, 15 cases with aneuploidies, three cases with mosaic aneuploidies, three cases with deletion/duplications, and one case with psu idic. Compared with KT, C-MoKa identified a number of additional chromosomal rearrangements (a diagnostic yield of 21.2% (44/208)). This superior resolution demonstrated direct clinical utility, as exemplified in cases A241520 and A241632 where it enabled detection of KT-missed cryptic SVs, effectively reducing false-negative rates (Fig. 6 and Supplementary Fig. 1). This observation validates the established capability of 3D genomics to reveal occult chromosomal rearrangements that previously undetectable by conventional cytogenetic methods [33]. Supplementary Fig. 2 displays characteristic C-MoKa profiles for multiple SVs and CNVs, including balanced translocations, Robertsonian translocations, inversions, deletions and duplications; detailed interpretation principles and full technical specifications of C-MoKa (covering resolution and sensitivity across distinct variant types) are provided in the supplementary materials.

For CNV-based platforms, we enhanced WES with supplementary copy number probes (~ 100 kb resolution) and complementarily performed CMA/WES in all samples for CA detection. Collectively, they obtained a combined diagnostic yield of 18.3% (38/208), therein 15 cases with aneuploidies, two cases with mosaic aneuploidies, 19 cases with deletion/duplications, two cases with ROHs. Additionally, eight cases with SNVs were identified by WES. Compared to CNV-based detection, C-MoKa serves as a more powerful tool for SV detection. WES independently identified eight SNVs and three sub-resolution deletions beyond C-MoKa’s detection capability. The combination of C-MoKa and WES enables comprehensive coverage of the genomic variation spectrum, and appears to yield the most promising diagnostic outcomes in prenatal diagnosis [34–36]. However, if only considering the CNV detection efficacy of WES or CMA, the combined use of C-MoKa and WES/CMA (22.6%) is already superior to the traditional KT + CNV combination scheme (20.7%). All SV events including balanced translocations, Robertsonian translocations, and inversions were detectable using C-MoKa, as illustrated in our retrospective cohort. According to published literatures [37–39], OGM has limitations in detecting Robertsonian translocations due to the presence of heterochromatin in related regions. C-MoKa shows distinct potential advantages in detecting such rearrangements; however, direct head-to-head performance comparison between C-MoKa and OGM was not conducted in this study, and further parallel cohort studies are needed for comprehensive evaluation.

We established a robust internal protocol for C-MoKa and determined a minimum cell threshold of 0.2 million for a successful assay. Compared to the conventional application of “KT + CNV” in prenatal diagnosis, “CNV + C-MoKa” might be the most effective technology combination that may achieve lower patient turnaround times and overall costs. According to current C-MoKa workflow in our prenatal scenario, the complete turnaround time from sample receipt to clinical report totals 10–11 days, substantially shorter than conventional KT (which typically requires 3–4 weeks), but a little longer than CMA (5–6 days) and WES (6–7 days) in most diagnostic laboratories owing to the extra cell culture step required for C-MoKa. Falling NGS costs set C-MoKa at ¥2,500 (~$350) per sample. Low pricing benefits from scaled sequencing, automated library preparation, streamlined bioinformatics and integrated multi-assay detection. It costs less than combined SOC tests and captures balanced/complex SVs undetectable by routine methods.

Interestingly, the cell culture process itself can influence the representation of CAs. This was exemplified by case A241079, where CMA on uncultured cells indicated a mosaic monosomy X (~ 60% mosaicism), whereas analyses on cultured cells (KT and C-MoKa) suggested a higher mosaicism rate (80%) or a non-mosaic monosomy X, respectively. This discrepancy underscores the potential impact of cell culture on mosaicism detection and highlights the critical need for standardized protocols to minimize interpretive artifacts [40, 41]. Future research should prioritize direct C-MoKa analysis of uncultured amniocytes to circumvent culture-induced artifacts that compromise diagnostic accuracy.

C-MoKa has limited sensitivity for low-level mosaicism below 20% under our current analytical pipeline and sequencing depth (~ 30 million read-pairs). However, we note that one mosaic monosomy X case with 80% mosaicism was successfully identified, indicating that detection sensitivity is likely dependent on both the proportion of abnormal cells and the specific type of chromosomal abnormality. For low-level mosaicism below this threshold, the spatial contact signal from the abnormal cell population may be diluted below the algorithm’s detection cutoff (observed/expected ratio ≥ 2 for SVs; contact intensity thresholds of 0.7/1.3 for CNVs). Regarding the impact of sequencing depth, our current standard of 30 million reads was empirically determined to balance cost and performance. The deeper sequencing (e.g., 50–60 million reads) might improve the signal-to-noise ratio for low-level mosaic calls, though this would increase turnaround time and cost. Future optimization studies are needed to establish a depth-sensitivity curve for different variant types. In our data, mosaic aneuploidies (e.g., trisomy 21 mosaicism) appeared more challenging for C-MoKa than balanced SVs, likely because whole-chromosome copy number changes produce more subtle 3D contact alterations than discrete breakpoints. For small CNVs (< 1 Mb) with low mosaicism, detection may be even more limited. While most prenatally detected low-level mosaic abnormalities are associated with favorable or uncertain outcomes, some (e.g., mosaic trisomy 13, 18, or monosomy X) may still carry significant risks. Therefore, when C-MoKa yields a negative result but ultrasound or other clinical findings raise suspicion for mosaicism, we recommend confirmatory testing with a more sensitive method such as FISH on uncultured cells or high-depth CNV-seq.

This study employs a two-phase design comprising retrospective validation and prospective clinical evaluation. The retrospective cohort with confirmed chromosomal abnormalities verifies the basic detection performance of C-MoKa, while the unselected prospective cohort simulates real clinical scenarios. This design effectively reduces selection bias, improves internal validation capability and enhances the generalizability of research results. However, the comparatively small sample size and lack of multicenter review are two of the study’s weaknesses. To validate the usefulness of C-MoKa, future research with larger sample sizes and multicenter clinical trials is preferred. Furthermore, satellite-repeat domains (e.g., pericentromeric/telomeric regions) exhibit intrinsic mappability constraints. Consequently, SVs occurring within these territories require orthogonal validation to ensure accurate interpretation.

## Conclusions

In conclusion, both retrospective and prospective cohort research demonstrate the potential utility of C-MoKa in prenatal diagnostic testing, exhibiting high concordance with SOC techniques. Compared to the conventional application of “KT + CNV” in prenatal diagnosis, “CNV + C-MoKa” might be the most effective combination that can map nearly all of genomic SVs and maximize diagnostic yield. However, clinical implementation of C-MoKa in routine prenatal diagnostics requires further validation through prospective multicenter studies with larger cohorts.

## Supplementary Information

Below is the link to the electronic supplementary material.

Supplementary Material 1

Supplementary Material 2

Supplementary Material 3

Supplementary Material 4

Supplementary Material 5

Supplementary Material 6

Supplementary Material 7

## Data Availability

The data and material will be shared on reasonable request to the corresponding author.

## Abbreviations

KT: Karyotyping
FISH: Fluorescence in situ hybridization
CMA: Chromosomal microarray analysis
CNV: Copy number variant
WES: Whole-exome sequencing
WGS: Whole-genome sequencing
SNV: Single-nucleotide variant
Indel: Insertion-deletion
SV: Structural variant
OGM: Optical genome mapping
RT: Robertsonian translocation
UPD: Uniparental disomy
SOC: Standard of care
AF: Amniotic fluid
CA: Chromosomal abnormality
IRB: Institutional Review Board
iWES: improved WES
