# Supplementary material for: Diagnostic evaluation and clinical utility of C-MoKa for prenatal genetic testing
Source: J Transl Med. 2026 Jul 14;24:1270. doi: 10.1186/s12967-026-08641-w (PMC13651082; doi:10.1186/s12967-026-08641-w)
Supplement: Supplementary file 1 [file 12967_2026_8641_MOESM4_ESM.pdf]

Supplementary Table 1. Chromosomal abnormabilities of each sample identified by C-MoKa and Karyotyping/CMA/iWES in the restrospective cohort

Sample ID	Age of pregnant woman	Clinical indication	C-MoKa		Karyotyping	CMA (hg37)	iWES (CNV)(GRCh37)	Clinical Outcome
			SV (GRCh37)	CNV (GRCh37)				
A240661	1994-03-14	NIPT: Sex chromosome abnormalities	Clinical significant variant not detected	seq[GRCh37] del(6)(p25.3p21.33)<br>chr6:g.180,001_31,380,000del (21%)	45,X[13]/46,XX[50]	Clinical significant variant not detected	Not applicable	Live birth, with minor malformations
A240686	1991-07-14	1q21.1-q21.2 4.55Mb microdeletion was detected in the couple's carrier screening	Clinical significant variant not detected	seq[GRCh37] del(16)(p12.3p12.2) chr16:g.21,140,001_21,380,000del	46,XY	arr[hg37] 16p12.3p12.2(21,151,611 -21,379,439)x1	Not applicable	Live birth
A240697	1997-01-07	NIPT: Sex chromosome abnormalities	Clinical significant variant not detected	seq[GRCh37] del(X)(q25q27.3)<br>chrX:g.127,740,001_146,420,000del	46,X,del(X)(q25q27)	arr[hg37] Xq25q27.3(127,776,026 -146,408,776)x1	Not applicable	Live birth
A240715	1995-10-22	1.The husband had a balanced translocation of chromosomes 3 and 4; 2.embryo arrest twice	46,XY,t(3;4)(q11.2;p15.31)<br>chr3:0-95700000 → chr4:19400000-0<br>chr3:198022430-95700000 → chr4:19400000-191154276	Clinical significant variant not detected	46,XY,t(3;4)（q12;p16)	Clinical significant variant not detected	Not applicable	Live birth, with minor malformations
A240820	1995-02-07	NIPT: High risk for trisomy 18	Clinical significant variant not detected	+18	47,XY,+18	arr[hg37] 18p11.32q23(136,228 -78,013,728)x3	Not applicable	Termination of pregnancy
A240822	1989-05-31	The pregnant woman herself had a balanced translocation of chromosomes	46,XY,t(1;13)(p32.3;q13.1)<br>chr13:0-32500000 → chr1:51600000-0<br>chr13:115169878-32500000 → chr1:51600000-249250621	Clinical significant variant not detected	46,XY,t(1;13)（p32;q12)	Clinical significant variant not detected	Not applicable	Live birth
A240825	2000-02-25	NT 3.5mm	45,XY,rob(14;22)(q10;q10)<br>chr22:51304566-14700000 → chr14:17600000-107349540	Clinical significant variant not detected	45,XY,der(14;22)(q10;q10)	Not applicable	Clinical significant variant not detected	Live birth
A240838	1986-10-17	Advanced maternal age	Clinical significantvariant not detected	seq[GRCh37] del(4)(q22.1)<br>chr4:g.88,520,001_89,120,000del	46,XX	arr[hg37] 4q22.1(88,540,983 -89,103,609)x1	Not applicable	Termination of pregnancy
A240839	1996-11-23	Chromosomal abnormalities in pregnant women	45,XY,rob(15;22)(q10;q10)<br>chr22:51304566-14700000 → chr15:19000000-102531392	Clinical significant variant not detected	45,XY,der(15;22)(q10;q10)	Not applicable	Clinical significant variant not detected	Termination of pregnancy
A240844	1985-11-01	1.Twin pregnancy; 2.This fetus had a varus foot; 3. Ventricular absence	Clinical significant variant not detected	+18	47,XY,+18	Not applicable	47,XY,+18	Live birth
A240877	1995-10-06	1. DS: 1/194; 2. NIPT: High risk of trisomy 18 and more number 10	Clinical significant variant not detected	mos(+10)(~45%),<br>mos(+18)(~55%)	47,XX,+18[60]/47,XX,+10[5]	arr[hg37] 10p15.3q26.3(100,047 -135,426,386)x2-3;<br>arr[hg37] 18p11.32q23(136,228 -78,013,728)x2-3	Not applicable	Termination of pregnancy
A240898	1998-10-06	NIPT: Sex chromosome abnormalities	Clinical significant variant not detected	+X	47,XXY	arr[hg37] Xp22.33q28(168,552 -155,233,098)x2	Not applicable	Termination of pregnancy
A240904	1988-11-16	1. Advanced maternal age; 2. Twin pregnancy; 3.Noninvasive abnormality	Clinical significant variant not detected	Clinical significant variant not detected	47,XY,+2[6]/46,XY[45]	Not applicable	Clinical significant variant not detected	Termination of pregnancy
A240918	1995-02-18	1.The husband had Robertsonian translocation of chromosome 13 and 14; 2. PGT	45,XX,rob(13;14)(q10;q10)<br>chr14:107349540-17600000 → chr13:17900000-115169878	Clinical significant variant not detected	45,XX,der(13;14)(q10;q10)	Clinical significant variant not detected	Not applicable	Live birth
A240938	1985-02-20	1. Advanced maternal age; 2.NIPT: Sex chromosome abnormalities	Clinical significant variant not detected	+X	47,XXX	arr[hg37] Xp22.33q28(168,552 -155,233,098)x2	Not applicable	Live birth
A240939	1996-11-10	NIPT： dup(18p22.1)-1.01Mb	Clinical significant variant not detected	seq[GRCh37] dup(18)(q22.1) chr18:g.65,460,001_66,440,000dup	46,XX	arr[hg37] 18q22.1(65,484,935 -66,432,957)x3	Not applicable	Live birth, with minor malformations
A240951	1989-09-09	1. Advanced maternal age; 2. NT 2.8mm	Clinical significant variant not detected	seq[GRCh37] dup(X)(p21.3p21.2)<br>chrX:g.29,160,001_29,900,000dup	46,XY	arr[hg37] Xp21.3p21.2(29,169,416 -29,880,574)x2	Not applicable	Live birth
A240965	1988-01-10	1. Advanced maternal age; 2. NIPT: Sex chromosome abnormalities	Clinical significant variant not detected	seq[GRCh37] dup(X)(p22.33)<br>chrX:g.140,001_2,680,000dup;<br>+Y	47,XYY	arr[hg37] Yp11.2q12(2,650,424 -28,799,654)x2	Not applicable	Termination of pregnancy
A240967	1990-04-18	High risk of DS	Clinical significant variant not detected	seq[GRCh37] del(7)(p22.1)<br>chr7:g.5,080,001_6,220,000del	46,XX	arr[hg37] 7p22.1(chr7:5,016,415 -6,223,478)x1	Not applicable	Termination of pregnancy
A240970	1984-07-26	Advanced maternal age	Clinical significant variant not detected	+X	47,XXY	arr[hg37] Xp22.33q28(168,552 -155,233,098)x2	Not applicable	Neonatal death
A240981	1999-11-01	1. NIPT: increased chromosome 8; 2. High risk of NTD	Clinical significant variant not detected	seq[GRCh37]<br>20p11.21(22500001_25500000)x2 hmz;<br>seq[GRCh37]<br>20q11.21q11.23(31500001_35000000)x2 hmz	46,XY	arr[hg37] 20p11.22q11.23(21,850,786 -36,153,360)x2 hmz	Not applicable	Termination of pregnancy
A240985	1995-05-25	NIPT：del（20q13.31-q13.33）6.76Mb；del（17q11.2-q12）5.93Mb	Clinical significant variant not detected	seq[GRCh37] del(X)(p22.31)<br>chrX:g.6,440,001_8,140,000del	46,XY	arr[hg37] Xp22.31(6,455,277 -8,135,645)x0	Not applicable	Live birth
A241004	1988-04-06	1. Advanced maternal age; 2. NIPT: del(9q22.1-q22.31,3.59Mb)-M	Clinical significant variant not detected	Clinical significant variant not detected	46,XY,del(2)(q31?)[7]/46,XY[63]	Clinical significant variant not detected	Not applicable	Live birth
A241040	1994-07-28	NIPT: a duplication of chromosome 13	Clinical significant variant not detected	seq[GRCh37] dup(13)(q12.12)<br>chr13:g.23,520,001_24,900,000dup;<br>seq[GRCh37] dup(22)(q12.3)<br>chr22:g.34,140,001_34,700,000dup	46,XY	arr[hg37] 13q12.12(23,499,836 -24,958,572)x3	Not applicable	Live birth
A241000	1988-12-10	1. Advanced maternal age; 2. NIPT: del(Xq21)	Clinical significant variant not detected	seq[GRCh37] del(X)(q21.1q21.2)<br>chrX:g.80,360,001_84,900,000del	46,XX	arr[hg37] Xq21.1q21.2(80,368,967 -84,889,120)x1	Not applicable	Live birth
A241058	1997-09-09	High risk of DS	Clinical significant variant not detected	seq[GRCh37] dup(X)(q23)<br>chrX:g.109,340,001_110,680,000dup	46,XY	arr[hg37] Xq23(109,365,183 -110,660,823)x2	Not applicable	Live birth
A241059	1997-11-04	NIPT: dup(22q11.21,3.17Mb) -M	Clinical significant variant not detected	seq[GRCh37] dup(22)(q11.21)<br>chr22:g.18,900,001_21,480,000dup	46,XY	arr[hg37] 22q11.21(18,976,690 -21,464,764)x3	Not applicable	Termination of pregnancy
A241066	1993-01-09	1. High risk of DS; 2. NIPT: High risk of ES	Clinical significant variant not detected	+18	47,XY,+18	arr[hg37] 18p11.32q23(136,228 -78,013,728)x3	Not applicable	Termination of pregnancy
A241075	1998-02-28	NIPT: dup(Xp11.4-p21.1), 7.14M	Clinical significant variant not detected	seq[GRCh37] del(16)(p12.2)<br>chr16:g.21,920,001_22,460,000del;<br>seq[GRCh37] dup(X)(p21.2p11.4)<br>chrX:g.31,480,001_38,640,000dup;<br>seq[GRCh37] dup(X)(q21.33)<br>chrX:g.95,360,001_96,360,000dup	46,XX	arr[hg37] Xp21.2p11.4(31,498,800 -38,624,555)x3;<br>arr[hg37] 16p12.2(21,841,354 -22,442,007)x1	Not applicable	Live birth

A241079	1989-10-19	Advanced maternal age	Clinical significant variant not detected	-X	45,X[32]/47,XXX[8]	arr[hg37] Xp22.33q28(168,552 -155,233,098)x1-2	Not applicable	Termination of pregnancy
A241081	1993-10-07	1. Abnormal ultrasound soft markers; 2. NIPT: Sex chromosome abnormalities	Clinical significant variant not detected	+X	47,XXY	arr[hg37] Xp22.33q28(168,552 -155,233,098)x2	Not applicable	Termination of pregnancy
A241088	2002-10-10	Abnormal childbearing	Clinical significant variant not detected	seq[GRCh37] dup(6)(q16.2q16.3)<br>chr6:g.100,260,001_103,180,000dup	46,XY	arr[hg37] 6q16.2q16.3(100,287,021 -103,168,448)x3	Not applicable	Termination of pregnancy
A241095	1989-06-05	1. Advanced maternal age; 2. Abnormal ultrasound; 3. NIPT: High risk of DS	Clinical significant variant not detected	+21	47,XY,+21	arr[hg37] 21q11.2q22.3(15,016,487 -48,093,361)x3	Not applicable	Termination of pregnancy
A241096	1996-07-06	NIPT: sex chromosome abnormality	Clinical significant variant not detected	+X	47,XXY	arr[hg37] Xp22.33q28(168,552 -155,233,098)x2	Not applicable	Termination of pregnancy
A241104	1997-07-10	Balanced translocation of chromosomes in pregnant women	46,XY,t(1;14)(p22.3;q31.1)<br>chr14:0-80400000 → chr1:88100000-0<br>chr14:107349540-80400000 → chr1:88100000-249250621	seq[GRCh37] del(16)(p12.2)<br>chr16:g.21,920,001_22,720,000del	46,XY,t(1;14)(p22;q24)	arr[hg37] 16p12.2(21,841,354 -22,441,367)x1	Not applicable	Live birth
A241107	1981-04-01	Advanced maternal age	Clinical significant variant not detected	seq[GRCh37]<br>3p14.2p12.3(60500001_76000000)x2 hmz	46,XY	arr[hg37] 3p14.2p12.3(60,661,991 -74,487,772)x2 hmz	Not applicable	Termination of pregnancy
A241123	1998-01-25	1, DS: 1/54; 2. +18: 1/70	Clinical significant variant not detected	seq[GRCh37] del(13)(q33.2q33.3)<br>chr13:g.106,260,001_109,540,000del	46,XY	arr[hg37] 13q33.2q33.3(106,287,955 -109,523,585)x1	Not applicable	Termination of pregnancy
A241125	2004-01-16	DS: 1/65	Clinical significant variant not detected	seq[GRCh37] dup(15)(q13.2q13.3)<br>chr15:g.31,120,001_32,460,000dup;<br>seq[GRCh37] dup(Y)(p11.31q11.221)<br>chrY:g.2,620,001_19,560,000dup (56%);<br>seq[GRCh37] del(Y)(q11.221q11.223)<br>chrY:g.19,540,001_24,520,000del	45,X[30]/46,X,psu,idic(Y)(q11.2)[26]/47,X,psu,idic(Y)(q11.2)x2[11]	arr[hg37] 15q13.2q13.3(31,098,691 -32,444,044)x3;<br>arr[hg37] Yq11.222q12(20,618,888 -28,799,654)x0;<br>arr[hg37] Yp11.2q11.222(2,650,425 -19,556,683)x2	Not applicable	Termination of pregnancy
A241130	1989-10-20	NIPT: High risk of DS	Clinical significant variant not detected	+21	47,XY,+21	arr[hg37] 21q11.2q22.3(15,016,487 -48,093,361)x3	Not applicable	Termination of pregnancy
A241131	1989-06-08	Advanced maternal age	Clinical significant variant not detected	seq[GRCh37] dup(2)(q34)<br>chr2:g.212,560,001_213,820,000dup	46,XY	arr[hg37] 2q34(211,749,185 -212,931,845)x3	Not applicable	Live birth, with minor malformations
A241134	1995-01-01	DS: 1/206	Clinical significant variant not detected	seq[GRCh37] dup(2)(p12)<br>chr2:g.78,580,001_79,980,000dup	46,XX	arr[hg37] 2p12(78,589,873 -79,995,493)x3	Not applicable	Live birth
A241172	1989-01-10	Chromosomal abnormalities in the husband	46,XX,t(11;15)(q23.3;q23)<br>chr11:0-116700000 → chr15:71700000-102531392<br>chr15:0-71700000 → chr11:116700000-135006516	Clinical significant variant not detected	46,XX,t(11;15)(q23;q24)	Clinical significant variant not detected	Not applicable	Live birth
A241177	1989-09-08	Advanced maternal age	Clinical significant variant not detected	seq[GRCh37] del(8)(q22.3) chr8:g.104,580,001_104,980,000del	46,XY	arr[hg37] 8q22.3(104,588,426 -104,962,406)x1	Not applicable	Termination of pregnancy
A241186	1997-01-15	Chromosome of husband: 46,X,Yqh+	Clinical significant variant not detected	seq[GRCh37] dup(8)(p23.2p23.1)<br>chr8:g.5,960,001_6,640,000dup	46,XY	arr[hg37] 8p23.2p23.1(5,982,476 -6,622,607)x3	Not applicable	Live birth
A241195	1988-11-27	Chromosome of husband:<br>46,XY,t(16;19)(p13.3p13.1)	Clinical significant variant not detected	seq[GRCh37] dup(1)(p31.1)<br>chr1:g.76,060,001_77,320,000dup	46,XX	arr[hg37] 1p31.1(76,101,656 -77,308,653)x3	Not applicable	Live birth
A241199	1992-09-02	NIPT: dup(13q31.3,1.22Mb) -M	Clinical significant variant not detected	seq[GRCh37] dup(13)(q31.3)<br>chr13:g.93,380,001_94,200,000dup	46,XY	arr[hg37] 13q31.3(93,380,513 -94,211,428)x3	Not applicable	Termination of pregnancy
A241206	1992-12-21	Chromosome of husband:<br>46, XY,t（1;4）(q43;p13)	46,XX,t(1;4)(q42.3;p11)<br>chr1:0-236000000 → chr4:48900000-0<br>chr1:249250621-236000000 → chr4:48900000-191154276	Clinical significant variant not detected	46,XX,t(1;4)(q43;p13)	Clinical significant variant not detected	Not applicable	Live birth
A241219	1989-06-12	1. Advanced maternal age; 2. Abnormal ultrasound; 3. One of the couples had chromosomal abnormalities	46,XX,inv(5)(q12.1q22.2)<br>chr5:0-62500000 → chr5:112300000-62500000 →<br>chr5:112300000-180915260	Clinical significant variant not detected	46,XX,inv(5)(q12q23)	Not applicable	Clinical significant variant not detected	Live birth, with minor malformations
A241220	1987-10-18	1. Advanced maternal age; 2. NIPT: increased number of sex chromosomes	Clinical significant variant not detected	+X	47,XXY	arr[hg37] Xp22.33q28(168,552 -155,233,098)x2	Not applicable	Live birth, with minor malformations
A241237	1993-10-11	NIPT: The number of sex chromosomes is low	Clinical significant variant not detected	Clinical significant variant not detected	45,X[8]/46,XX[46]	arr[hg37] Xp22.33q28(168,552 -155,233,098)x1-2	Not applicable	Live birth
A241260	1993-02-12	High-risk Pregnancy surveillance	Clinical significant variant not detected	mos(-X)(19.7%)	45,X[11]/46,XX[39]	arr[hg37] Xp22.33q28(168,552 -155,233,098)x1-2	Not applicable	Termination of pregnancy
A241265	1992-10-30	high risk of trisomy 13	Clinical significant variant not detected	mos(+13)(28%)	47,XY,+13[31]/46,XY[15]	arr[hg37] 13q11q34(19,436,286 -115,107,733)x2-3	Not applicable	Termination of pregnancy
A241302	1998-08-04	high risk of trisomy 18	Clinical significant variant not detected	mos(+18)(46%)	47,XY,+18[31]/46,XY[23]	arr[hg37] 18p11.32q23(136,228 -78,013,728)x2-3	Not applicable	Termination of pregnancy
A241306	1998-07-24	1. High risk of NT; 2. NIPT: High risk of ES	46,XY,der(13)t(13;18)(q34;q12.1)<br>chr13:0-114500000 → chr18:31400000-78077248	seq[GRCh37] del(13)(q34)<br>chr13:g.114,460,001_115,120,000del,<br>seq[GRCh37] dup(18)(q12.1q23)<br>chr18:g.31,380,001_78,040,000dup	46,XY,der(13)t(13;18)(qter;q12)	Not applicable	Seq[hg38]dup(18)(q12.1q23)chr 18:g.31422551_78016654dup	Termination of pregnancy
A241320	1990-03-19	1. Chromosome of the pregnant woman : 45,XX, rob (14; 21) (q10;q10); 2. PGT	45,XX,rob(14;21)(q10;q10)	Clinical significant variant not detected	45,XX,der(14;21)(q10;q10)	Clinical significant variant not detected	Not applicable	Live birth, with minor malformations
A241330	1999-10-23	1. DS:1/98; 2. The fetal sacrococcygeal region was not clearly visualized	46,XX,t(10;19)(q22.3;p12)<br>chr10:0-78500000 → chr19:21200000-0<br>chr10:135534747-78500000 → chr19:21200000-59128983	Clinical significant variant not detected	46,XX,t(10;19)(q22;p13.1)	Clinical significant variant not detected	Not applicable	Live birth
