# Supplementary material for: Diagnostic evaluation and clinical utility of C-MoKa for prenatal genetic testing
Source: J Transl Med. 2026 Jul 14;24:1270. doi: 10.1186/s12967-026-08641-w (PMC13651082; doi:10.1186/s12967-026-08641-w)
Supplement: Supplementary file 2 [file 12967_2026_8641_MOESM5_ESM.pdf]

Supplementary Table 2. Chromosomal abnormabilities of each sample identified by C-MoKa and Karyotyping/CMA/iWES in the prospective cohort

Sample ID	Age of pregnant woman	Clinical indication	C-MoKa		Karyotyping	CMA (hg37)	iWES CNV (GRCh37)	iWES SNV (GRCh37)	Clinical Outcome
			SV (GRCh37)	CNV (GRCh37)					
A241331	1994-11-13	PGT	Clinical significant variant not detected	Clinical significant variant not detected	46,XX	Not applicable	Clinical significant variant not detected	Clinical significant variant not detected	Live birth
A241332	1987-09-30	Abnormal childbearing	Clinical significant variant not detected	Clinical significant variant not detected	46,XX	Not applicable	Clinical significant variant not detected	Clinical significant variant not detected	Live birth
A241333	1986-01-01	1. Advanced maternal age; 2. High risk of NT	Clinical significant variant not detected	seq[GRCh37] del(22)(q13.1) chr22:g.40,440,001_40,540,000del	46,XY	Not applicable	Clinical significant variant not detected	Clinical significant variant not detected	Live birth
A241334	1989-04-24	NIPT: del(5q21.3,3.43M)-M	Clinical significant variant not detected	Clinical significant variant not detected	46,XX	Not applicable	Clinical significant variant not detected	Clinical significant variant not detected	Live birth
A241335	1986-11-19	1. Advanced maternal age; 2. High risk of NT	Clinical significant variant not detected	Clinical significant variant not detected	46,XY	Not applicable	Clinical significant variant not detected	Clinical significant variant not detected	Live birth
A241336	1994-02-12	Thalassemia in both spouses	Clinical significant variant not detected	Clinical significant variant not detected	46,XX	Not applicable	Seq[hg37]del(16)(p13.3p13.3) chr16:g.215996_231107	Clinical significant variant not detected	Termination of pregnancy
A241337	1984-06-02	1. Advanced maternal age; 2. Abnormal childbearing	Clinical significant variant not detected	Clinical significant variant not detected	46,XX	Not applicable	Clinical significant variant not detected	Clinical significant variant not detected	Live birth
A241338	1990-11-04	Abnormal childbearing	Clinical significant variant not detected	Clinical significant variant not detected	46,XX	Clinical significant variant not detected	Not applicable	Not applicable	Live birth
A241339	1985-12-08	Advanced maternal age	Clinical significant variant not detected	Clinical significant variant not detected	46,XX	Not applicable	Clinical significant variant not detected	Clinical significant variant not detected	Live birth
A241340	1989-06-06	1. Advanced maternal age; 2. NIPT: The number of chromosome 15 was monosomy	Clinical significant variant not detected	Clinical significant variant not detected	46,XX	Not applicable	Clinical significant variant not detected	Clinical significant variant not detected	Live birth
A241341	1994-03-20	The husband had a history of heredity	Clinical significant variant not detected	Clinical significant variant not detected	46,XY	Not applicable	Clinical significant variant not detected	*GJB2* :c.235del(p.Leu79CysfsTer3, hom	Termination of pregnancy
A241342	1994-08-03	NIPT: dup(18q12.3,1.01Mb)-M	Clinical significant variant not detected	Clinical significant variant not detected	46,XX	Clinical significant variant not detected	Not applicable	Not applicable	Live birth
A241343	1995-11-10	1. High Risk of DS; 2. Abnormal ultrasound	Clinical significant variant not detected	Clinical significant variant not detected	46,XY	Not applicable	Clinical significant variant not detected	Clinical significant variant not detected	Live birth
A241344	1996-06-27	Abnormal ultrasound	Clinical significant variant not detected	seq[GRCh37] del(4)(p16.3) chr4:g.80,001_3,780,000del	46,XX	Not applicable	Seq[hg38]del(4)(p16.3p16.3)chr4:g.85730_3766106del	Clinical significant variant not detected	Termination of pregnancy
A241345	1991-09-22	Abnormal ultrasound	Clinical significant variant not detected	Clinical significant variant not detected	46,XX	Clinical significant variant not detected	Not applicable	Not applicable	Live birth
A241346	1986-10-13	1. Advanced maternal age; 2. High Risk of DS; 3. High Risk of ES	Clinical significant variant not detected	+18	47,XY,+18	arr[hg37] 18p11.32q23(136,228-78,013,728)x3	Not applicable	Not applicable	Termination of pregnancy
A241347	1987-04-21	1. Advanced maternal age; 2. NIPT: del(13q31.1,1.41Mb)-F	Clinical significant variant not detected	Clinical significant variant not detected	46,XY	Clinical significant variant not detected	Not applicable	Not applicable	Termination of pregnancy
A241348	1984-10-26	Advanced maternal age	Clinical significant variant not detected	Clinical significant variant not detected	46,XX	Clinical significant variant not detected	Not applicable	Not applicable	Live birth
A241350	1995-10-19	Abnormal childbearing	Clinical significant variant not detected	Clinical significant variant not detected	46,XX	Not applicable	Clinical significant variant not detected	Clinical significant variant not detected	Live birth
A241351	1995-12-16	Abnormal ultrasound soft markers	Clinical significant variant not detected	Clinical significant variant not detected	46,XX	Not applicable	Clinical significant variant not detected	*PKD1* : c.7927C>T(p.Arg2643Cys)	Live birth, with minor malformations
A241352	1985-09-18	NIPT: del(4q28.3,3.6M)-M, T7:7.575,3.058	Clinical significant variant not detected	Clinical significant variant not detected	46,XY	Not applicable	Clinical significant variant not detected	Clinical significant variant not detected	Live birth
A241353	1994-08-10	Abnormal ultrasound	Clinical significant variant not detected	Clinical significant variant not detected	46,XX	Not applicable	Clinical significant variant not detected	*ACTB* : c.848T>C(p.Met283Thr)	Termination of pregnancy
A241354	1996-02-24	Abnormal childbearing	Clinical significant variant not detected	Clinical significant variant not detected	46,XX	Not applicable	Clinical significant variant not detected	Clinical significant variant not detected	Live birth
A241355	2000-10-06	1. Twin pregnancy; 2. Abnormal ultrasound	Clinical significant variant not detected	Clinical significant variant not detected	46,XY	Not applicable	Clinical significant variant not detected	Clinical significant variant not detected	Live birth
A241356	2000-10-06	1. Twin pregnancy; 2. Abnormal ultrasound	Clinical significant variant not detected	Clinical significant variant not detected	46,XY	Not applicable	Clinical significant variant not detected	Clinical significant variant not detected	Live birth
A241357	1996-04-10	1.Abnormal ultrasound; 2. Abnormal childbearing; 3. High Risk of NT	Clinical significant variant not detected	seq[GRCh37] dup(21)(q11.2q22.3) chr21:g.15,420,001_48,120,000dup (21.6%)	47,XY,+21[12]/46,XY[28]	Not applicable	47,XY,+21 (15%)	Clinical significant variant not detected	Termination of pregnancy
A241358	2002-07-29	The pregnant woman herself is mentally retarded	Clinical significant variant not detected	seq[GRCh37] del(7)(q11.23) chr7:g.72,720,001_74,160,000del	46,XX	arr[hg37] 7q11.23(72,650,209-74,207,613)x1	Not applicable	Not applicable	Termination of pregnancy
A241359	1991-12-11	1. Abnormal ultrasound; 2. Abnormal childbearing	Clinical significant variant not detected	Clinical significant variant not detected	46,XY	Not applicable	Clinical significant variant not detected	Clinical significant variant not detected	Live birth

A241360	1995-05-21	1. Twin pregnancy; 2. Abnormal ultrasound	Clinical significant variant not detected	Clinical significant variant not detected	46,XX	Not applicable	Clinical significant variant not detected	Clinical significant variant not detected	Live birth
A241361	1995-05-21	1. Twin pregnancy; 2. Abnormal ultrasound	Clinical significant variant not detected	Clinical significant variant not detected	46,XY	Not applicable	Not applicable	Not applicable	Live birth
A241362	1999-01-06	Abnormal childbearing	Clinical significant variant not detected	Clinical significant variant not detected	46,XY	Clinical significant variant not detected	Not applicable	Not applicable	Termination of pregnancy
A241363	1991-03-16	1. Twin pregnancy; 2. Abnormal ultrasound	Clinical significant variant not detected	Clinical significant variant not detected	46,XX	Not applicable	Clinical significant variant not detected	Clinical significant variant not detected	Live birth
A241364	1991-03-16	1. Twin pregnancy; 2. Abnormal ultrasound	Clinical significant variant not detected	Clinical significant variant not detected	46,XX	Not applicable	Clinical significant variant not detected	Clinical significant variant not detected	Live birth
A241365	1999-12-26	Abnormal ultrasound	Clinical significant variant not detected	Clinical significant variant not detected	46,XY	Clinical significant variant not detected	Not applicable	Not applicable	Live birth
A241366	1977-08-04	Advanced maternal age	Clinical significant variant not detected	Clinical significant variant not detected	46,XY	Clinical significant variant not detected	Not applicable	Not applicable	Live birth
A241367	1986-11-29	1. Advanced maternal age; 2. High risk of DS	Clinical significant variant not detected	+21	47,XY,+21	arr[hg37] 21q11.2q22.3(15,016,487 -48,093,361)x3	Not applicable	Not applicable	Termination of pregnancy
A241368	1983-11-29	Advanced maternal age	Clinical significant variant not detected	Clinical significant variant not detected	46,XY	Not applicable	Clinical significant variant not detected	Clinical significant variant not detected	Live birth
A241369	2000-05-13	NIPT: the high risk for trisomy 13	Clinical significant variant not detected	Clinical significant variant not detected	46,XY	Clinical significant variant not detected	Not applicable	Not applicable	Live birth
A241370	1990-02-18	Noninvasive failure	Clinical significant variant not detected	seq[GRCh37] dup(18)(p11.32p11.1) chr18:g.380,001_15,440,000dup; seq[GRCh37] del(18)(p11.32) chr18:g.120,001_400,000del	46,XY,dup(18)(p11.21p11.32)	Not applicable	Seq[hg38]dup(18)(p11.32p11.21)chr18:g.361924_15381135dup; Seq[hg38]del(18)(p11.32p11.32)chr18:g.10500_361424del	Clinical significant variant not detected	Termination of pregnancy
A241371	1996-06-08	NIPT: T10:6.799,4.084	Clinical significant variant not detected	Clinical significant variant not detected	46,XX	Clinical significant variant not detected	Not applicable	Not applicable	Live birth
A241372	1993-05-19	1. High risk of DS; 2. Abnormal childbearing	Clinical significant variant not detected	Clinical significant variant not detected	46,XX	Not applicable	Clinical significant variant not detected	Clinical significant variant not detected	Live birth
A241373	1995-10-19	High risk of NT	Clinical significant variant not detected	Clinical significant variant not detected	46,XY	Not applicable	Clinical significant variant not detected	Clinical significant variant not detected	Termination of pregnancy
A241374	1994-10-26	Abnormal childbearing	Clinical significant variant not detected	Clinical significant variant not detected	46,XX	Not applicable	Clinical significant variant not detected	Clinical significant variant not detected	Live birth
A241375	1987-06-16	1. Advanced maternal age; 2. Abnormal childbearing	Clinical significant variant not detected	Clinical significant variant not detected	46,XY	Clinical significant variant not detected	Not applicable	Not applicable	Live birth
A241376	1994-02-25	Abnormal childbearing	Clinical significant variant not detected	Clinical significant variant not detected	46,XY	Not applicable	Clinical significant variant not detected	Clinical significant variant not detected	Live birth
A241377	1984-04-22	1. Advanced maternal age; 2. Abnormal childbearing	Clinical significant variant not detected	Clinical significant variant not detected	46,XY	Clinical significant variant not detected	Not applicable	Not applicable	Live birth
A241378	2001-03-05	1. PGT; 2. CMA:del(10p15.1p14,4.85Mb) and del(14q12,1.59Mb) in her husband's peripheral blood	Clinical significant variant not detected	seq[GRCh37] dup(6)(q11.1q13) chr6:g.61,920,001_70,380,000dup(33%)	46,XY	Not applicable	Clinical significant variant not detected	Clinical significant variant not detected	Live birth
A241379	1981-03-20	Abnormal ultrasound	Clinical significant variant not detected	mos(+X)(28.8%)	46,XX	Clinical significant variant not detected	Not applicable	Not applicable	Live birth
A241380	2000-07-31	NIPT: dup(1p12-p13.3,9.97Mb)	Clinical significant variant not detected	seq[GRCh37] dup(7)(q11.21q11.23) chr7:g.66,800,001_73,480,000dup(37%)	46,XY	arr[hg37]7q11.21(62,569,502 -66,791,947)x2 hmz	Not applicable	Not applicable	Live birth
A241381	1987-08-13	1.Advanced maternal age; 2.Abnormal ultrasound soft markers	Clinical significant variant not detected	Clinical significant variant not detected	46,XY	Clinical significant variant not detected	Not applicable	Not applicable	Live birth
A241382	2001-03-16	High risk of DS	Clinical significant variant not detected	Clinical significant variant not detected	46,XX	Clinical significant variant not detected	Not applicable	Not applicable	Termination of pregnancy
A241383	1995-07-09	NIPT: dup(10q22.3-q23.31,8.61Mb)-M	Clinical significant variant not detected	seq[GRCh37] dup(10)(q22.3q23.31) chr10:g.81,220,001_89,640,000dup	46,XY	Not applicable	Seq[hg37]dup(10)(q22.3q23.31)chr10:g.81255561_89624305dup	Clinical significant variant not detected	Live birth
A241384	1989-03-29	1. Advanced maternal age; 2. NIPT: 47, XXY	Clinical significant variant not detected	+X	47,XXY	arr[hg37] Xp22.33q28(168,552 -155,233,098)x2	Not applicable	Not applicable	Live birth
A241385	1996-08-18	Abnormal ultrasound	Clinical significant variant not detected	Clinical significant variant not detected	46,XY	Not applicable	Clinical significant variant not detected	Clinical significant variant not detected	Live birth, with major malformations
A241386	1994-10-03	Abnormal ultrasound	Clinical significant variant not detected	Clinical significant variant not detected	46,XY	Not applicable	Clinical significant variant not detected	Clinical significant variant not detected	Live birth
A241387	1993-10-21	Noninvasive other abnormalities	Clinical significant variant not detected	Clinical significant variant not detected	46,XY	Clinical significant variant not detected	Not applicable	Not applicable	Live birth
A241388	1993-05-02	1. High risk of NT, 2. NIPT: T7:19.449,11.824	Clinical significant variant not detected	Clinical significant variant not detected	46,XY	Clinical significant variant not detected	Not applicable	Not applicable	Live birth

A241389	1988-12-18	1.Advanced maternal age; 2.Abnormal ultrasound	Clinical significant variant not detected	Clinical significant variant not detected	46,XX	Not applicable	Clinical significant variant not detected	*PKD2* : c.1365G>A(p.Trp455Ter)	Live birth
A241390	1995-01-25	High risk of DS	Clinical significant variant not detected	Clinical significant variant not detected	46,XY	Clinical significant variant not detected	Not applicable	Not applicable	Live birth
A241391	1996-12-06	Abnormal ultrasound	Clinical significant variant not detected	Clinical significant variant not detected	46,XY	Not applicable	Clinical significant variant not detected	Clinical significant variant not detected	Live birth
A241392	1993-11-07	1. Abnormal ultrasound; 2. Abnormal childbearing	Clinical significant variant not detected	Clinical significant variant not detected	46,XX	Not applicable	Clinical significant variant not detected	Clinical significant variant not detected	Live birth
A241393	1990-12-21	1. Abnormal ultrasound; 2. NIPT: High risk for trisomy 13	Clinical significant variant not detected	Clinical significant variant not detected	46,XX	Not applicable	Clinical significant variant not detected	Clinical significant variant not detected	Live birth
A241394	1986-10-19	Advanced maternal age; 2.Abnormal childbearing	Clinical significant variant not detected	Clinical significant variant not detected	46,XX	Not applicable	Clinical significant variant not detected	Clinical significant variant not detected	Termination of pregnancy
A241395	1991-01-10	NIPT: dup(11q12.2-q25,73.16Mb)	Clinical significant variant not detected	Clinical significant variant not detected	46,XX	Not applicable	Clinical significant variant not detected	Clinical significant variant not detected	Live birth
A241396	1988-10-21	1. Advanced maternal age; 2. One of the couples had chromosomal abnormalities	Clinical significant variant not detected	Clinical significant variant not detected	46,XY	Clinical significant variant not detected	Not applicable	Not applicable	Live birth
A241397	1989-10-10	1. Advanced maternal age; 2. Abnormal childbearing	Clinical significant variant not detected	Clinical significant variant not detected	46,XY	Clinical significant variant not detected	Not applicable	Not applicable	Live birth
A241398	1997-11-18	Abnormal childbearing	Clinical significant variant not detected	seq[GRCh37] del(2)(q22.1q22.2) chr2:g.139,700,001_142,700,000del	46,XY	arr[hg37] 2q22.1q22.2(139,724,301-142,685,234)x1	Not applicable	Not applicable	Live birth
A241399	1984-09-14	Advanced maternal age	Clinical significant variant not detected	Clinical significant variant not detected	46,XX	Clinical significant variant not detected	Not applicable	Not applicable	Live birth
A241400	1993-08-02	NIPT: dup(14q31.1-q31.2,5.56Mb)	Clinical significant variant not detected	Clinical significant variant not detected	46,XX	Not applicable	Clinical significant variant not detected	Clinical significant variant not detected	Live birth
A241401	2006-05-28	High risk of DS	Clinical significant variant not detected	Clinical significant variant not detected	46,XY	Clinical significant variant not detected	Not applicable	Not applicable	Termination of pregnancy
A241402	1993-04-22	1. Abnormal ultrasound; 2.High risk of NT	Clinical significant variant not detected	+21	47,XY,+21	Not applicable	Seq[hg38]dup(21)(p12q22.3)chr21:g.5011798_46699483dup	Clinical significant variant not detected	Termination of pregnancy
A241403	1995-05-18	Abnormal ultrasound	Clinical significant variant not detected	Clinical significant variant not detected	46,XX	Not applicable	Clinical significant variant not detected	Clinical significant variant not detected	Live birth
A241404	1996-09-30	Abnormal ultrasound	Clinical significant variant not detected	Clinical significant variant not detected	46,XX	Not applicable	Clinical significant variant not detected	Clinical significant variant not detected	Live birth
A241405	1991-08-06	1. Abnormal ultrasound; 2. Abnormal childbearing	Clinical significant variant not detected	Clinical significant variant not detected	46,XY	Not applicable	Clinical significant variant not detected	Clinical significant variant not detected	Live birth
A241406	1981-12-12	Advanced maternal age	Clinical significant variant not detected	Clinical significant variant not detected	46,XY	Clinical significant variant not detected	Not applicable	Not applicable	Live birth
A241407	1987-04-04	Advanced maternal age	Clinical significant variant not detected	Clinical significant variant not detected	46,XX	Clinical significant variant not detected	Not applicable	Not applicable	Live birth, with minor malformations
A241408	1996-01-13	NIPT: dup(20p12.1-p12.3,7.18Mb)	Clinical significant variant not detected	Clinical significant variant not detected	46,XX	Clinical significant variant not detected	Not applicable	Not applicable	Live birth
A241409	1998-03-22	High risk of DS	Clinical significant variant not detected	seq[GRCh37] dup(1)(q44) chr1:g.245,140,001_246,740,000dup	46,XX	arr[hg37] 1q44(245,169,309-246,709,272)x3	Not applicable	Not applicable	Live birth
A241410	1984-07-15	Advanced maternal age	Clinical significant variant not detected	Clinical significant variant not detected	46,XX	Clinical significant variant not detected	Not applicable	Not applicable	Termination of pregnancy
A241411	1982-07-10	Advanced maternal age	Clinical significant variant not detected	Clinical significant variant not detected	46,XY	Clinical significant variant not detected	Not applicable	Not applicable	Live birth
A241412	1988-03-05	1. Advanced maternal age; 2. Abnormal childbearing; 3.Abnormal ultrasound	Clinical significant variant not detected	Clinical significant variant not detected	46,XX	Not applicable	Clinical significant variant not detected	Clinical significant variant not detected	Live birth
A241413	1997-12-23	NIPT: dup(18p11.23-p11.31,1.01Mb)-M	Clinical significant variant not detected	Clinical significant variant not detected	46,XY	Not applicable	Clinical significant variant not detected	Clinical significant variant not detected	Live birth
A241414	1994-10-20	Abnormal childbearing	Clinical significant variant not detected	Clinical significant variant not detected	46,XX	Not applicable	Clinical significant variant not detected	Clinical significant variant not detected	Live birth
A241415	1990-09-12	High risk of NTD	Clinical significant variant not detected	mos(+12)(25.1%)	47,XX,+12[11]/46,XX[39]	Not applicable	47,XY,+12 (20%)	Clinical significant variant not detected	Termination of pregnancy
A241416	1998-10-28	Abnormal ultrasound	Clinical significant variant not detected	Clinical significant variant not detected	46,XY	Not applicable	Clinical significant variant not detected	Clinical significant variant not detected	Termination of pregnancy
A241417	1996-05-12	NIPT: T7:7.463,4.355;T14:7.962,5.657	Clinical significant variant not detected	Clinical significant variant not detected	46,XX	Clinical significant variant not detected	Not applicable	Not applicable	Live birth

A241418	1993-09-10	NIPT: dup(7p21.3,4.15Mb)-M	Clinical significant variant not detected	Clinical significant variant not detected	46,XX	Not applicable	Clinical significant variant not detected	Clinical significant variant not detected	Live birth
A241419	1984-02-13	1. Advanced maternal age; 2. Abnormal ultrasound	Clinical significant variant not detected	Clinical significant variant not detected	46,XX	Not applicable	Clinical significant variant not detected	Clinical significant variant not detected	Termination of pregnancy
A241420	1997-01-25	Abnormal ultrasound	Clinical significant variant not detected	Clinical significant variant not detected	46,XX	Not applicable	Clinical significant variant not detected	Clinical significant variant not detected	Live birth
A241421	1991-04-13	The carrier screened positive	Clinical significant variant not detected	Clinical significant variant not detected	46,XY	Not applicable	Seq[hg38]del(X)(q28q28)chrX:g.154152939_154289751del	Clinical significant variant not detected	Live birth
A241422	1997-02-20	Abnormal ultrasound	Clinical significant variant not detected	Clinical significant variant not detected	46,XX	Not applicable	Clinical significant variant not detected	*GALC* : c.19/1T>C(p.Leu634Ser)	Termination of pregnancy
A241423	1997-08-15	NIPT: del(Xp11.4-p22.33,39.47Mb)-M	Clinical significant variant not detected	Clinical significant variant not detected	46,XY	Clinical significant variant not detected	Not applicable	Not applicable	Live birth
A241424	1991-09-04	1. Abnormal childbearing; 2.Abnormal ultrasound	Clinical significant variant not detected	Clinical significant variant not detected	46,XY	Not applicable	Clinical significant variant not detected	Clinical significant variant not detected	Live birth
A241425	1987-04-13	1. Advanced maternal age; 2. Abnormal childbearing; 3. High risk of NT	Clinical significant variant not detected	Clinical significant variant not detected	46,XY	Not applicable	Clinical significant variant not detected	Clinical significant variant not detected	Live birth
A241426	1994-06-27	High risk of NT	Clinical significant variant not detected	Clinical significant variant not detected	46,XX	Not applicable	Clinical significant variant not detected	Clinical significant variant not detected	Live birth
A241427	1988-12-03	Advanced maternal age	Clinical significant variant not detected	Clinical significant variant not detected	46,XX	Clinical significant variant not detected	Not applicable	Not applicable	Live birth
A241428	1979-09-05	Advanced maternal age	Clinical significant variant not detected	Clinical significant variant not detected	46,XX	Clinical significant variant not detected	Not applicable	Not applicable	Live birth
A241429	1995-10-25	High risk of DS	Clinical significant variant not detected	Clinical significant variant not detected	46,XX	Clinical significant variant not detected	Not applicable	Not applicable	Live birth
A241430	1989-04-10	Abnormal childbearing	Clinical significant variant not detected	Clinical significant variant not detected	46,XY	Clinical significant variant not detected	Clinical significant variant not detected	Clinical significant variant not detected	Live birth
A241431	2000-05-15	Maternal chromosome: 46, XX, inv(14)(p11.1p12)	Clinical significant variant not detected	Clinical significant variant not detected	46,XY	Clinical significant variant not detected	Not applicable	Not applicable	Termination of pregnancy
A241432	1994-04-29	Abnormal childbearing	Clinical significant variant not detected	Clinical significant variant not detected	46,XY	Clinical significant variant not detected	Not applicable	Not applicable	Live birth
A241433	1995-02-20	Abnormal childbearing	Clinical significant variant not detected	Clinical significant variant not detected	46,XX	Not applicable	Clinical significant variant not detected	Clinical significant variant not detected	Live birth
A241434	1995-03-24	High risk of NT	Clinical significant variant not detected	Clinical significant variant not detected	46,XY	Not applicable	Clinical significant variant not detected	Clinical significant variant not detected	Live birth
A241435	1995-05-03	Abnormal childbearing	Clinical significant variant not detected	Clinical significant variant not detected	46,XY	Clinical significant variant not detected	Not applicable	Not applicable	Live birth
A241436	1993-01-26	Abnormal ultrasound	Clinical significant variant not detected	Clinical significant variant not detected	46,XY	Not applicable	Clinical significant variant not detected	Clinical significant variant not detected	Live birth
A241437	2000-07-06	1. High risk of DS; 2. NIPT: 45,X	Clinical significant variant not detected	Clinical significant variant not detected	46,XX	Clinical significant variant not detected	Not applicable	Not applicable	Termination of pregnancy
A241438	1995-09-13	High risk of DS	Clinical significant variant not detected	Clinical significant variant not detected	46,XX	Clinical significant variant not detected	Not applicable	Not applicable	Live birth
A241439	1997-11-21	Abnormal ultrasound	Clinical significant variant not detected	Clinical significant variant not detected	46,XY	Not applicable	Clinical significant variant not detected	Clinical significant variant not detected	Live birth
A241440	1988-12-27	NIPT: High risk for trisomy 13	Clinical significant variant not detected	Clinical significant variant not detected	46,XX	Clinical significant variant not detected	Not applicable	Not applicable	Termination of pregnancy
A241441	1993-02-19	Abnormal ultrasound	Clinical significant variant not detected	Clinical significant variant not detected	46,XY	Clinical significant variant not detected	Not applicable	Not applicable	Live birth
A241442	1987-09-07	NIPT: Chromosome 1p36 deletion syndrome	Clinical significant variant not detected	Clinical significant variant not detected	46,XX	Clinical significant variant not detected	Not applicable	Not applicable	Live birth
A241443	1995-02-01	Abnormal ultrasound	Clinical significant variant not detected	Clinical significant variant not detected	46,XY	Not applicable	Clinical significant variant not detected	Clinical significant variant not detected	Live birth
A241444	1996-01-09	NNIPT:dup(7q31.1,3.97Mb)-M	Clinical significant variant not detected	Clinical significant variant not detected	46,XY	Not applicable	Clinical significant variant not detected	Clinical significant variant not detected	Live birth
A241445	1989-04-01	1. Advanced maternal age, 2. Abnormal ultrasound soft markers	Clinical significant variant not detected	Clinical significant variant not detected	46,XX	Not applicable	Clinical significant variant not detected	Clinical significant variant not detected	Live birth
A241446	1989-09-24	1.Advanced maternal age; 2. Noninvasive other abnormalities	Clinical significant variant not detected	Clinical significant variant not detected	46,XY	Clinical significant variant not detected	Not applicable	Not applicable	Live birth
A241447	1987-02-02	1.Advanced maternal age; 2.High risk of NT	Clinical significant variant not detected	Clinical significant variant not detected	46,XY	Not applicable	Clinical significant variant not detected	*PTPN11* : c.188A>G(p.Tyr63Cys)	Termination of pregnancy
A241448	1993-02-28	Abnormal ultrasound	Clinical significant variant not detected	Clinical significant variant not detected	46,XY	Not applicable	Clinical significant variant not detected	Clinical significant variant not detected	Live birth

A241449	1996-06-18	Abnormal ultrasound	Clinical significant variant not detected	Clinical significant variant not detected	46,XY	Not applicable	Clinical significant variant not detected	Clinical significant variant not detected	Live birth
A241450	2002-11-05	1. Abnormal ultrasound soft markers; 2.Abnormal ultrasound	Clinical significant variant not detected	Clinical significant variant not detected	46,XX	Not applicable	Clinical significant variant not detected	*ACTB* : c.547C>T(p.Arg183Trp)	Termination of pregnancy
A241451	1983-08-02	Advanced maternal age	Clinical significant variant not detected	Clinical significant variant not detected	46,XY	Clinical significant variant not detected	Not applicable	Not applicable	Termination of pregnancy
A241452	1994-03-03	Abnormal ultrasound	Clinical significant variant not detected	Clinical significant variant not detected	46,XX	Not applicable	Clinical significant variant not detected	Clinical significant variant not detected	Live birth
A241453	1986-10-15	Advanced maternal age	Clinical significant variant not detected	Clinical significant variant not detected	46,XY	Clinical significant variant not detected	Not applicable	Not applicable	Live birth
A241454	1988-07-28	1. Abnormal childbearing; 2.Abnormal ultrasound	Clinical significant variant not detected	Clinical significant variant not detected	46,XX	Not applicable	Clinical significant variant not detected	Clinical significant variant not detected	Live birth
A241455	1991-10-19	Abnormal childbearing	Clinical significant variant not detected	Clinical significant variant not detected	46,XX	Not applicable	Clinical significant variant not detected	Clinical significant variant not detected	Live birth
A241456	1994-11-14	Abnormal ultrasound	Clinical significant variant not detected	Clinical significant variant not detected	46,XY	Not applicable	Clinical significant variant not detected	Clinical significant variant not detected	Live birth
A241457	1998-08-24	Abnormal ultrasound	Clinical significant variant not detected	Clinical significant variant not detected	46,XX	Not applicable	Clinical significant variant not detected	Clinical significant variant not detected	Live birth
A241458	1999-11-23	Abnormal ultrasound	Clinical significant variant not detected	Clinical significant variant not detected	46,XX	Not applicable	Clinical significant variant not detected	Clinical significant variant not detected	Live birth
A241460	1999-08-25	Abnormal ultrasound	Clinical significant variant not detected	Clinical significant variant not detected	46,XX	Not applicable	Clinical significant variant not detected	Clinical significant variant not detected	Termination of pregnancy
A241461	1982-07-20	Advanced maternal age	Clinical significant variant not detected	Clinical significant variant not detected	46,XX	Clinical significant variant not detected	Not applicable	Not applicable	Termination of pregnancy
A241462	2002-06-20	NIPT: dup(8p23.1,3.89Mb)-M	Clinical significant variant not detected	Clinical significant variant not detected	46,XY	Clinical significant variant not detected	Not applicable	Not applicable	Termination of pregnancy
A241463	1994-08-16	Cousins of both spouses, genetic abnormalities	Clinical significant variant not detected	ROH	46,XX	Not applicable	ROH	Clinical significant variant not detected	Live birth
A241464	1998-10-01	Abnormal ultrasound	Clinical significant variant not detected	Clinical significant variant not detected	46,XX	Not applicable	Clinical significant variant not detected	Clinical significant variant not detected	Live birth
A241465	1986-01-05	1. Advanced maternal age; 2.NIPT: 47,XXX	Clinical significant variant not detected	+X	47,XXX	arr[hg37] Xp22.33q28(168,552 - 155,233,098)x2	Not applicable	Not applicable	Termination of pregnancy
A241466	1994-08-04	Abnormal ultrasound	Clinical significant variant not detected	Clinical significant variant not detected	46,XY	Not applicable	Clinical significant variant not detected	Clinical significant variant not detected	Live birth
A241468	1999-04-23	NIPT: Angelman syndrome /Prader-Willi syndrome	Clinical significant variant not detected	seq[GRCh37] del(15)(q11.2q13.1) chr15:g.23,680,001_28,540,000del	46,XY	Not applicable	Seq[hg38]del(15)(q11.2q13.1)chr15:g.23358471_28579278del	Clinical significant variant not detected	Termination of pregnancy
A241469	1990-03-27	Abnormal ultrasound soft markers	Clinical significant variant not detected	Clinical significant variant not detected	46,XX	Not applicable	Clinical significant variant not detected	Clinical significant variant not detected	Live birth
A241470	1988-09-14	Advanced maternal age	Clinical significant variant not detected	+X	47,XXY	arr[hg37] Xp22.33q28(168,552 - 155,233,098)x2	Not applicable	Not applicable	Termination of pregnancy
A241471	1989-10-18	1. Advanced maternal age; 2.Maternal t(1;14); 3.Three generations of preimplantation syncytial embryos	Clinical significant variant not detected	Clinical significant variant not detected	46,XY	Clinical significant variant not detected	Not applicable	Not applicable	Termination of pregnancy
A241472	1994-05-11	Abnormal childbearing	Clinical significant variant not detected	Clinical significant variant not detected	46,XX	Clinical significant variant not detected	Not applicable	Not applicable	Live birth
A241473	1993-04-06	1.High Risk of DS; 2. .NIPT: T7:6.772,4.4	Clinical significant variant not detected	Clinical significant variant not detected	46,XY	Not applicable	Clinical significant variant not detected	Clinical significant variant not detected	Termination of pregnancy
A241474	1992-03-09	Abnormal childbearing	Clinical significant variant not detected	Clinical significant variant not detected	46,XX	Clinical significant variant not detected	Not applicable	Not applicable	Termination of pregnancy
A241475	1990-10-08	Abnormal childbearing	Clinical significant variant not detected	Clinical significant variant not detected	46,XX	Not applicable	Clinical significant variant not detected	Clinical significant variant not detected	Live birth
A241476	1987-02-04	1. Advanced maternal age, 2. Abnormal childbearing	Clinical significant variant not detected	Clinical significant variant not detected	46,XX	Not applicable	Clinical significant variant not detected	Clinical significant variant not detected	Live birth
A241477	1995-07-27	High risk of ES	Clinical significant variant not detected	+18	47,XX,+18	arr[hg37] 18p11.32q23(136,228 - 78,013,728)x3	Not applicable	Not applicable	Termination of pregnancy
A241478	1987-06-10	1. Advanced maternal age; 2. Abnormal childbearing	Clinical significant variant not detected	seq[GRCh37] dup(X)(p22.33p22.32) chrX:g.3,600,001_5,600,000dup	46,XX	arr[hg37] Xp22.33p22.32(3,605,919 - 5,678,167)x3	Not applicable	Not applicable	Live birth
A241479	1988-02-22	1. Advanced maternal age; 2. Abnormal childbearing; 3.Abnormal ultrasound	Clinical significant variant not detected	Clinical significant variant not detected	46,XY	Not applicable	Clinical significant variant not detected	Clinical significant variant not detected	Live birth

A241480	2002-05-22	NIPT: The number of sex chromosomes is low	Clinical significant variant not detected	-X	45,X	arr[hg37] Xp22.33q28(168,552 - 155,233,098)x2	Not applicable	Not applicable	Termination of pregnancy
A241481	1991-08-11	Abnormal ultrasound	Clinical significant variant not detected	Clinical significant variant not detected	46,XX	Not applicable	Clinical significant variant not detected	Clinical significant variant not detected	Termination of pregnancy
A241482	1994-08-08	1. Abnormal ultrasound; 2. Abnormal childbearing	Clinical significant variant not detected	Clinical significant variant not detected	46,XY	Not applicable	Clinical significant variant not detected	Clinical significant variant not detected	Live birth
A241483	1993-08-14	Abnormal ultrasound	Clinical significant variant not detected	Clinical significant variant not detected	46,XY	Not applicable	Clinical significant variant not detected	Clinical significant variant not detected	Termination of pregnancy
A241484	1988-12-12	1. Advanced maternal age; 2.NIPT: 45,X	Clinical significant variant not detected	Clinical significant variant not detected	46,XX	Clinical significant variant not detected	Not applicable	Not applicable	Live birth
A241485	1998-12-30	Abnormal ultrasound	Clinical significant variant not detected	Clinical significant variant not detected	46,XX	Not applicable	Clinical significant variant not detected	Clinical significant variant not detected	Live birth
A241486	2005-11-04	NIPT:del(13q12.12,1.52 Mb)-F	Clinical significant variant not detected	Clinical significant variant not detected	46,XX	Clinical significant variant not detected	Not applicable	Not applicable	Termination of pregnancy
A241487	1999-09-06	1.High Risk of DS; 2. Abnormal ultrasound; 3.NIPT: High risk for trisomy 13; T16:30.008.24.103	Clinical significant variant not detected	Clinical significant variant not detected	46,XX	Clinical significant variant not detected	Not applicable	Not applicable	Termination of pregnancy
A241488	1986-08-23	Advanced maternal age	Clinical significant variant not detected	Clinical significant variant not detected	46,XY	Not applicable	Clinical significant variant not detected	Clinical significant variant not detected	Live birth
A241489	1987-01-01	1. Advanced maternal age, 2. Abnormal childbearing	Clinical significant variant not detected	Clinical significant variant not detected	46,XX	Clinical significant variant not detected	Not applicable	Not applicable	Live birth
A241490	1991-11-25	NIPT: Hhigh risk of DS	Clinical significant variant not detected	+21	47,XX,+21	arr[hg37] 21q11.2q22.3(15,016,487 - 48,093,361)x3	Not applicable	Not applicable	Termination of pregnancy
A241491	1989-02-12	1. Advanced maternal age; 2. High Risk of DS	Clinical significant variant not detected	+21	47,XY,+21	arr[hg37] 21q11.2q22.3(15,016,487 - 48,093,361)x3	Not applicable	Not applicable	Termination of pregnancy
A241492	1984-08-28	Advanced maternal age	Clinical significant variant not detected	Clinical significant variant not detected	46,XY	Clinical significant variant not detected	Not applicable	Not applicable	Live birth
A241493	2003-05-20	Abnormal childbearing	Clinical significant variant not detected	Clinical significant variant not detected	46,XY	Not applicable	Seq[hg37]del(16)(p13.3p13.3)chr16:g.215996_231107del	Clinical significant variant not detected	Termination of pregnancy
A241494	1986-01-05	NIPT: High risk of ES	Clinical significant variant not detected	+18	47,XX,+18	arr[hg37] 18p11.32q23(136,228 - 78,013,728)x3	Not applicable	Not applicable	Termination of pregnancy
A241495	1997-07-21	NIPT: 45,X	Clinical significant variant not detected	Clinical significant variant not detected	46,XY	Clinical significant variant not detected	Not applicable	Not applicable	Live birth
A241496	1989-11-24	Advanced maternal age	Clinical significant variant not detected	Clinical significant variant not detected	46,XX	Clinical significant variant not detected	Not applicable	Not applicable	Live birth
A241497	1992-05-16	1. High Risk of DS; 2. Abnormal ultrasound	Clinical significant variant not detected	Clinical significant variant not detected	46,XY	Not applicable	Clinical significant variant not detected	Clinical significant variant not detected	Live birth
A241498	1993-09-19	High Risk of DS	Clinical significant variant not detected	Clinical significant variant not detected	46,XY	Clinical significant variant not detected	Not applicable	Not applicable	Termination of pregnancy
A241499	1997-01-14	Abnormal ultrasound	Clinical significant variant not detected	seq[GRCh37] del(15)(q11.2q13.1) chr15:g.22,780,001_28,540,000del	46,XY	Not applicable	Seq[hg37]del(15)(q11.2q13.1)chr15:g.23182327_28955752del	Clinical significant variant not detected	Termination of pregnancy
A241500	1991-10-11	1. Abnormal ultrasound; 2. Abnormal childbearing	Clinical significant variant not detected	Clinical significant variant not detected	46,XY	Not applicable	Clinical significant variant not detected	Clinical significant variant not detected	Live birth
A241501	1986-05-01	Advanced maternal age	Clinical significant variant not detected	mos(-21)(21.3%)	46,XX	Not applicable	Clinical significant variant not detected	Clinical significant variant not detected	Termination of pregnancy
A241502	1992-03-08	High Risk of DS	Clinical significant variant not detected	Clinical significant variant not detected	46,XY	Not applicable	Clinical significant variant not detected	Clinical significant variant not detected	Live birth
A241503	1990-02-01	High Risk of DS	Clinical significant variant not detected	Clinical significant variant not detected	46,XY	Clinical significant variant not detected	Not applicable	Not applicable	Live birth
A241504	1989-10-03	1. Advanced maternal age; 2. Abnormal ultrasound	Clinical significant variant not detected	+21	47,XX,+21	Not applicable	47,XX,+21	Clinical significant variant not detected	Termination of pregnancy
A241505	1988-05-04	1. Advanced maternal age; 2. Abnormal ultrasound	Clinical significant variant not detected	Clinical significant variant not detected	46,XX	Not applicable	Clinical significant variant not detected	Clinical significant variant not detected	Live birth
A241506	1998-10-20	High Risk of DS	Clinical significant variant not detected	Clinical significant variant not detected	46,XX	Clinical significant variant not detected	Not applicable	Not applicable	Live birth
A241507	1995-01-06	Abnormal ultrasound	Clinical significant variant not detected	Clinical significant variant not detected	46,XX	Not applicable	Clinical significant variant not detected	Clinical significant variant not detected	Live birth
A241509	1985-04-19	1. Advanced maternal age; 2. Abnormal ultrasound	Clinical significant variant not detected	Clinical significant variant not detected	46,XX	Not applicable	Clinical significant variant not detected	*ACTB* : c.633C>G(p.Asp211Glu)	Termination of pregnancy

A241510	1997-04-08	NIPT: del(5p14.1-p15.2,15.16Mb)	Clinical significant variant not detected	seq[GRCh37] del(5)(p15.2p14.1) chr5:g.13,200,001_28,200,000del	46,XX,del(5)(p14p15.2)	arr[hg37] 5p15.2p14.1(13,205,466-28,195,328)x1	Not applicable	Not applicable	Termination of pregnancy
A241511	1993-08-12	NIPT: The number of chromosome 7 was monosomy	Clinical significant variant not detected	Clinical significant variant not detected	46,XY	Not applicable	Clinical significant variant not detected	Clinical significant variant not detected	Live birth
A241512	1994-01-07	NIPT: 45,X	Clinical significant variant not detected	mos(-X)(27.3%)	45,X[20]/46,XX[35]	Clinical significant variant not detected	Not applicable	Not applicable	Termination of pregnancy
A241513	1994-08-05	1. High Risk of NTD; 2. NIPT:T7:68.280,38.379	Clinical significant variant not detected	Clinical significant variant not detected	46,XX	Not applicable	Clinical significant variant not detected	Clinical significant variant not detected	Live birth
A241514	1989-05-10	Advanced maternal age	Clinical significant variant not detected	Clinical significant variant not detected	46,XY	Clinical significant variant not detected	Not applicable	Not applicable	Live birth
A241515	1983-05-20	Advanced maternal age	Clinical significant variant not detected	Clinical significant variant not detected	46,XX	Clinical significant variant not detected	Not applicable	Not applicable	Live birth
A241516	1987-06-12	1. Advanced maternal age; 2. Abnormal childbearing	Clinical significant variant not detected	+X	47,XXY	Not applicable	47,XXY	Clinical significant variant not detected	Termination of pregnancy
A241517	1993-11-14	NIPT: increased number of sex chromosomes	Clinical significant variant not detected	+Y	47,XYY	arr[hg37] Yp11.2q12(2,650,424-28,799,654)x2	Not applicable	Not applicable	Live birth
A241518	1997-11-01	1. High Risk of DS; 2. NIPT: 45,X	Clinical significant variant not detected	Clinical significant variant not detected	46,XX	Clinical significant variant not detected	Not applicable	Not applicable	Live birth
A241519	1983-02-11	1. Advanced maternal age; 2. Abnormal ultrasound	Clinical significant variant not detected	+21	47,XX,+21	Not applicable	47,XX,+21	Clinical significant variant not detected	Termination of pregnancy
A241520	2003-01-24	NIPT: T7:21.387,8.995	46,XX,inv(20)(p12.3p12.1) chr20:0-7700000 → chr20:12900000-7700000 → chr20:12900000-63025520	Clinical significant variant not detected	46,XX	Not applicable	Clinical significant variant not detected	Clinical significant variant not detected	Termination of pregnancy
A241521	1990-06-15	NIPT: Trisomy 9	Clinical significant variant not detected	Clinical significant variant not detected	46,XX	Clinical significant variant not detected	Not applicable	Not applicable	Live birth
A241522	1989-10-10	1. Advanced maternal age; 2. Abnormal ultrasound	Clinical significant variant not detected	Clinical significant variant not detected	46,XX	Not applicable	Clinical significant variant not detected	Clinical significant variant not detected	Live birth
A241525	1985-10-06	1. Advanced maternal age; 2. Abnormal ultrasound soft markers; 3. NIPT: dup(21q21.2,1.3Mb)-M	Clinical significant variant not detected	Clinical significant variant not detected	46,XX	Clinical significant variant not detected	Not applicable	Not applicable	Live birth
A241526	1998-04-05	Abnormal ultrasound	Clinical significant variant not detected	Clinical significant variant not detected	46,XX	Not applicable	Clinical significant variant not detected	Clinical significant variant not detected	Live birth
A241527	2001-06-17	Abnormal ultrasound	Clinical significant variant not detected	Clinical significant variant not detected	46,XY	Not applicable	Clinical significant variant not detected	Clinical significant variant not detected	Live birth
A241531	1987-10-07	1. Advanced maternal age, 2. NIPT: The number of chromosome 16 was monosomy	Clinical significant variant not detected	Clinical significant variant not detected	46,XY	Clinical significant variant not detected	Not applicable	Not applicable	Live birth
A241532	2003-01-29	NIPT: dup(18q22.1-q22.2,1.21Mb)-M;dup(9q34.2-q34.3,4.03Mb)-M	Clinical significant variant not detected	seq[GRCh37] dup(18)(q22.2) chr18:g.67,060,001_67,720,000dup	46,XX	arr[hg37] 18q22.2(66,799,758-67,729,387)x3	Not applicable	Not applicable	Termination of pregnancy
A241533	1990-08-21	NIPT: dup(10q21.1,3.25Mb)-M	Clinical significant variant not detected	seq[GRCh37] dup(10)(q21.1) chr10:g.54,480,001_57,720,000dup	46,XX	Not applicable	Seq[hg37]dup(10)(q21.1q21.1)chr10:g.54457525_57750045dup	Clinical significant variant not detected	Live birth
A241534	1990-04-17	Abnormal childbearing	46,XX,t(8;10)(q13.2;q21.1) chr8:0-68900000 → chr10:57500000-135534747 chr10:0-57500000 → chr8:68900000-146364022	Clinical significant variant not detected	46,XX,t(8;10)(q13;q21)	Not applicable	Clinical significant variant not detected	Clinical significant variant not detected	Live birth
A241535	2001-04-25	NIPT: dup(18q21.32-q21.33,1.01Mb)-M	Clinical significant variant not detected	seq[GRCh37] dup(18)(q21.32q21.33) chr18:g.58,540,001_59,400,000dup	46,XY	arr[hg37] 18q21.32q21.33(58,531,717-59,377,674)x3	Not applicable	Not applicable	Live birth
A241536	1995-09-28	Abnormal ultrasound	Clinical significant variant not detected	Clinical significant variant not detected	46,XY	Not applicable	Clinical significant variant not detected	Clinical significant variant not detected	Live birth
A241537	1994-03-04	NIPT: dup(3p11.1-p12.2,8.12Mb)-M	Clinical significant variant not detected	seq[GRCh37] dup(3)(p12.2p12.1) chr3:g.82,340,001_84,800,000dup seq[GRCh37] dup(3)(p12.1p11.1) chr3:g.86,320,001_90,220,000dup	46,XY	arr[hg37] 3p12.1p11.1(86,344,433-90,485,635)x3	Not applicable	Not applicable	Live birth
A241538	1991-01-01	Abnormal childbearing	Clinical significant variant not detected	Clinical significant variant not detected	46,XX	Not applicable	Clinical significant variant not detected	Clinical significant variant not detected	Live birth

A241632	1989-01-31	1. Advanced maternal age; 2. Abnormal ultrasound soft markers	46,XY,der(2)(7qter->7q21.11::2p12->2qter),der(7)(7pter->7q21.11::2p16.3->2p12::2p16.3->2pter) chr7:159138663-78400000 → chr2:78200000-243199373 chr7:0-78400000 → chr2:50900000-78200000 → chr2:50900000-0	Clinical significant variant not detected	46,XY,?inv(2)(p23p13)	Clinical significant variant not detected	Not applicable	Not applicable	Live birth
A241672	1997-07-20	NIPT: del(Xp22.13-p22.2,6.82Mb);del(Xp11.3-p11.4,6.16Mb);del(Xq25-q26.3,5.2Mb)-M(XML)	Clinical significant variant not detected	Clinical significant variant not detected	46,XY,t(2;3;4)?(q31;q21;q21)[10]/46,XY[74]	Clinical significant variant not detected	Not applicable	Not applicable	Live birth
A241689	1993-06-16	NIPT: dup(15q11.2-q13.1,5.43Mb)	Clinical significant variant not detected	seq[GRCh37] dup(15)(q11.2q13.1) chr15:g.22,780,001_28,540,000dup	47,XX,+psu idic(15)(q13)	Not applicable	Seq[hg37]dup(15)(q11.2q13.1) chr15:g.23372396_28623371dup	Clinical significant variant not detected	Termination of pregnancy
A241708	1995-03-30	One of the couples had chromosomal abnormalities	46,XY,t(6;17)(q22.31;p11.2) chr6:0-121600000 → chr17:16500000-0 chr6:171115067-121600000 → chr17:16500000-81195210	Clinical significant variant not detected	46,XY,t(6;17)(q23;p12)	Clinical significant variant not detected	Not applicable	Not applicable	Live birth
A241728	2002-08-18	Abnormal ultrasound	Clinical significant variant not detected	seq[GRCh37] dup(8)(p22p21.2) chr8:g.14,100,001_24,240,000dup seq[GRCh37] dup(8)(q13.2q21.12) chr8:g.69,220,001_78,360,000dup seq[GRCh37] dup(8)(q21.13q23.2) chr8:g.83,960,001_111,380,000dup	46,XX,dup(8)(q13q23)	Not applicable	Seq[hg37]dup(8)(p22p21.2)chr8:g.14181611_24211940dup; Seq[hg37]dup(8)(q13.2q21.13) chr8:g.69243247_78360808dup; Seq[hg37]dup(8)(q21.13q23.2) chr8:g.83958538_111358098dup	Clinical significant variant not detected	Termination of pregnancy
A241867	1986-07-14	Advanced maternal age	Clinical significant variant not detected	Clinical significant variant not detected	46,XY	Clinical significant variant not detected	Not applicable	Not applicable	Live birth
A241868	1986-07-13	1. Advanced maternal age; 2. Abnormal childbearing	Clinical significant variant not detected	Clinical significant variant not detected	46,XY	Clinical significant variant not detected	Not applicable	Not applicable	Termination of pregnancy
A241871	1986-06-15	1. Advanced maternal age; 2. Abnormal childbearing	Clinical significant variant not detected	Clinical significant variant not detected	46,XY	Clinical significant variant not detected	Not applicable	Not applicable	Live birth
A241872	1987-11-23	Advanced maternal age	Clinical significant variant not detected	Clinical significant variant not detected	46,XY	Clinical significant variant not detected	Not applicable	Not applicable	Live birth
