# Supplementary material for: Diagnostic evaluation and clinical utility of C-MoKa for prenatal genetic testing
Source: J Transl Med. 2026 Jul 14;24:1270. doi: 10.1186/s12967-026-08641-w (PMC13651082; doi:10.1186/s12967-026-08641-w)
Supplement: Supplementary file 3 [file 12967_2026_8641_MOESM6_ESM.pdf]

Supplementary Table 3. Validation of Discordance identified by C-MoKa and Karyotyping/CMA/iWES

Sample ID	C-MoKa		Karyotyping	CMA (hg37)	iWES CNV (GRCh37)	Validation		
	SV (GRCh37)	CNV (GRCh37)				Method	Result	Concordance
A240661	/		45,X[13]/46,XX[50]	Clinical significant variant not detected	Not applicable	STR	mosaic 45,X (10%)	in concordance with Karyotyping
		seq[GRCh37] del(6)(p25.3p21.33) chr6:g.180,001_31,380,000del(21%)				CNV-seq	not detected*	/
A240904	/	/	47,XY,+2[6]/46,XY[45]	Not applicable	Clinical significant variant not detected	STR	mosaic 47,XY,+2 (11%)	
A240965	/	seq[GRCh37] dup(X)(p22.33) chrX:g.140,001_2,680,000dup, +Y	47,XYY	arr[hg37] Yp11.2q12(2,650,424-28,799,654)x2	Not applicable	CNV-seq	seq[GRCh37] dup(X)(p22.33) chrX:g.140,001_2,680,000dup;+Y	in concordance with C-MoKa
A241004	/	/	46,XY,del(2)(q31?)[7]/46,XY[63]	Clinical significant variant not detected	Not applicable	STR	mosaic 46,XY,del(q31?)(10%)	
A241040	/	seq[GRCh37] dup(13)(q12.12) chr13:g.23,520,001_24,900,000dup, seq[GRCh37] dup(22)(q12.3) chr22:g.34,140,001_34,700,000dup	46,XY	arr[hg37] 13q12.12(23,499,836-24,958,572)x3	Not applicable	CNV-seq	seq[GRCh37] dup(13)(q12.12) chr13:g.23,520,001_24,900,000dup; seq[GRCh37] dup(22)(q12.3) chr22:g.34,160,001_34,720,000dup	in concordance with C-MoKa
A241075	/	seq[GRCh37] del(16)(p12.2) chr16:g.21,920,001_22,460,000del, seq[GRCh37] dup(X)(p21.2p11.4) chrX:g.31,480,001_38,640,000dup, seq[GRCh37] dup(X)(q21.33) chrX:g.95,360,001_96,360,000dup	46,XX	arr[hg37] Xp21.2p11.4(31,498,800-38,624,555)x3; arr[hg37] 16p12.2(21,841,354-22,442,007)x1	Not applicable	CNV-seq	seq[GRCh37] dup(X)(p21.2p11.4) chrX:g.31,460,001_38,520,000dup; seq[GRCh37] dup(X)(q21.33) chrX:g.95,560,001_95,900,000dup	in concordance with C-MoKa
A241079	/	-X	45,X[32]/47,XXX[8]	arr[hg37] Xp22.33q28(168,552-155,233,098)x1-2	Not applicable	STR	mosaic 45,X (65%)	in concordance with Karyotyping
A241125	/	seq[GRCh37] dup(15)(q13.2q13.3) chr15:g.31,120,001_32,460,000dup, mos seq[GRCh37] dup(Y)(p11.31q11.221) chrY:g.2,620,001_19,560,000dup(56%), seq[GRCh37] del(Y)(q11.221q11.223) chrY:g.19,540,001_24,520,000del	45,X[30]/46,X,psu,idic(Y)(q11.2)[26]/47,X,psu,idic(Y)(q11.2)x2[11]	arr[hg37] 15q13.2q13.3(31,098,691-32,444,044)x3; arr[hg37] Yq11.222q12(20,618,888-28,799,654)x0; arr[hg37] Yp11.2q11.222(2,650,425-19,556,683)x2	Not applicable	FISH	NA	
A241237	/	/	45,X[8]/46,XX[46]	arr[hg37] Xp22.33q28(168,552-155,233,098)x1-2	Not applicable	STR	mosaic 45,X (40%)	in concordance with Karyotyping
A241306	46,XY,der(13)t(13;18)(q34;q12.1) chr13:0-114500000 → chr18:31400000-78077248	seq[GRCh37] del(13)(q34) chr13:g.114,460,001_115,120,000del, seq[GRCh37] dup(18)(q12.1q23) chr18:g.31,380,001_78,040,000dup	46,XY,der(13)t(13;18)(qter;q12)	Not applicable	Seq[hg38]dup(18)(q12.1q23)chr18:g.31422551_78016654dup	CNV-seq	seq[GRCh37] del(13)(q34) chr13:g.114,480,001_115,120,000del; seq[GRCh37] dup(18)(q12.1q23) chr18:g.31,360,001_78,040,000dup	in concordance with C-MoKa
A241333	/	seq[GRCh37] del(22)(q13.1) chr22:g.40,440,001_40,540,000del	46,XY	Not applicable	Clinical significant variant not detected	CNV-seq	not detected#	/
A241378	/	seq[GRCh37] dup(6)(q11.1q13) chr6:g.61,920,001_70,380,000dup(33%)	46,XY	Not applicable	Clinical significant variant not detected	CNV-seq	not detected#	/
A241379	/	mos(+X)(28.8%)	46,XX	Not applicable	Not applicable	CNV-seq	not detected#	/
A241380	/	seq[GRCh37] dup(7)(q11.21q11.23) chr7:g.66,800,001_73,480,000dup(37%)	46,XY	arr[hg37] 7q11.21(62,569,502-66,791,947)x2 hmz	Not applicable	CNV-seq	not detected#	/
A241501	/	mos(-21)(21.3%)	46,XX	Not applicable	Clinical significant variant not detected	CNV-seq	not detected*	/
A241520	46,XX,inv(20)(p12.3p12.1) chr20:0-7700000 → chr20:12900000-7700000 → chr20:12900000-63025520	/	46,XX	Not applicable	Clinical significant variant not detected	FISH	46,XX,inv(20)(p12.3p12.1	in concordance with C-MoKa

A241632	46,XY,der(2)(7qter->7q21.11::2p12->2qter),der(7)(7pter->7q21.11::2p16.3->2p12::2p16.3->2pter) chr7:159138663-78400000 → chr2:78200000-243199373 chr7:0-78400000 → chr2:50900000-78200000 → chr2:50900000-0	/	46,XY,?inv(2)(p23p13)	Not applicable	Not applicable	FISH	46,XY,der(7)t(2;7)(p12;q21.11)inv(2)(p16.3p12)	in concordance with C-MoKa
A241672	/	/	46,XY,t(2;3;4)?(q31;q21;q21)[10]/46,XY[74]	Not applicable	Not applicable	FISH	NA	
A241689	/	seq[GRCh37] dup(15)(q11.2q13.1) chr15:g.22,780,001_28,540,000dup	47,XX,+psu idic(15)(q13)	Not applicable	Seq[hg37]dup(15)(q11.2q13.1) chr15:g.23372396_28623371dup	FISH	NA	

*, indicated that the relevant CNV with a low mosaic ratio was not detected in CNV-seq.

#, indicated that the relevant CNV was not detected in CNV-seq, the sequncing data of each sample was poorly qualified due to insufficient amniotic fluid sample and low DNA library concentration.
