# Supplementary material for: Diagnostic evaluation and clinical utility of C-MoKa for prenatal genetic testing
Source: J Transl Med. 2026 Jul 14;24:1270. doi: 10.1186/s12967-026-08641-w (PMC13651082; doi:10.1186/s12967-026-08641-w)
Supplement: Supplementary file 4 [file 12967_2026_8641_MOESM7_ESM.pdf]

Supplementary Table 4. Diagnostic performance metrics of C-MoKa for different variant types (including 95% confidence intervals)

Variation type	Sensitivity (%) [95% CI]	Specificity (%) [95% CI]	Positive predictive value (PPV) (%) [95% CI]	Negative predictive value (NPV) (%) [95% CI]	True positive (TP)	False positive (FP)	True negative (TN)	False negative (FN)
SVs	100.0 (51.0-100.0)	100.0 (98.2-100.0)	100.0 (51.0-100.0)	100.0 (98.2-100.0)	4	0	204	0
CNVs	84.2 (62.4-94.5)	99.5 (97.1-99.9)	94.1 (73.0-99.0)	98.4 (95.5-99.5)	16	1	188	3
Aneuploidies	100.0 (79.6-100.0)	100.0 (98.0-100.0)	100.0 (79.6-100.0)	100.0 (98.0-100.0)	15	0	193	0
Mosaicism	75.0 (30.1-95.4)	98.0 (95.1-99.2)	42.9 (15.8-75.0)	99.5 (97.2-99.9)	3	4	201	1
ROH	50.0 (9.5-90.5)	100.0 (98.2-100.0)	100.0 (20.7-100.0)	99.5 (97.3-99.9)	1	0	206	1
Overall	88.6 (76.0-95.0)	99.5 (98.8-99.8)	88.6 (76.0-95.0)	99.5 (98.8-99.8)	39	5	992	5
