# Supplementary material for: Diagnostic evaluation and clinical utility of C-MoKa for prenatal genetic testing
Source: J Transl Med. 2026 Jul 14;24:1270. doi: 10.1186/s12967-026-08641-w (PMC13651082; doi:10.1186/s12967-026-08641-w)
Supplement: Supplementary file 5 [file 12967_2026_8641_MOESM3_ESM.docx]

**Supplement Information for “Diagnostic Evaluation and Clinical Utility of C-MoKa for Prenatal Genetic Testing”**

This file has been provided by the authors to give readers additional information about the work.

1. **Principles of C-MoKa for Detecting Diverse Chromosomal SVs and CNVs**

C-MoKa converts the three-dimensional (3D) folding of the genome into a two-dimensional (2D) spatial contact matrix to extract the misconnection patterns of chromosomal fragments from the positions and orientations of abnormal corner signals in the matrix and deduce structural variants from the extracted signals. Briefly, FA-fixed proteins capture spatially proximal DNA fragments (the closer they are in space, the higher the capture probability) and sequencing reads are mapped to a two‑dimensional spatial contact matrix, where the X and Y axes represent genomic coordinates, and the colour intensity reflects contact frequency. In a normal linear genome, chromosomes display continuous signals along the diagonal of the matrix. When a structural variant occurs, previously distant segments become spatially adjacent, producing abnormally high‑signal spots (corner signals or off-diagonal peaks) in specific regions of the contact matrix. Interpreting abnormal signals requires two key pieces of information: breakpoint positions (the coordinates of the signal in the matrix) and signal orientation (the four corners of the matrix). Different combinations of positions and orientations correspond to different chromosomal connection structures.

Balanced translocation is a structural variation (SV) characterized by the reciprocal exchange of segments between two non‑homologous chromosomes, with no net loss of genetic material. In the contact matrix, strong cross‑chromosomal signals appear at the breakpoints of the two chromosomes, forming an intersecting cross‑like pattern. A symmetrical “cross‑shaped” hotspot is observed between the breakpoints on the two chromosomes, and the signal orientations on either side of the breakpoints exhibit diagonally complementary, as depicted in the newly added Supplementary Figure 2A.

Robertsonian Translocation is a special type of balanced translocation in which the entire long arms of two acrocentric chromosomes fuse at the centromere, accompanied by the loss of the short arms. Because the fusion spans the full long arms, the resulting signal is no longer a punctate hotspot but rather a broad, continuous, block‑like region of high intensity that connects the long arms of both chromosomes. Consequently, between the two acrocentric chromosomes, a dense, continuous high‑signal band extends from the centromere to the distal ends of the long arms, whereas the corresponding short‑arm regions exhibit no signal, reflecting the absence of material exchange and the exclusive involvement of the long arms in the fusion, as shown in Supplementary Figure 2B.

Inversion is a structural variation in which a segment within a single chromosome is flipped by 180° (i.e., its orientation is reversed). As a result of this reversal, the originally adjacent linear regions are no longer in proximity; instead, the outer flanks of the two breakpoints become spatially close to the inverted ends of the segment. Signals are detected exclusively between two different positions on the same chromosome, and the connection orientation is opposite to the linear reference, meaning that the off‑diagonal signal shift in the contact matrix is mirror‑symmetric with respect to the normal diagonal (Supplementary Figure 2C).

For CNV analysis, we integrated CNV sequencing data with the spatial contact matrix. The final CNV calls were derived from overlapping regions identified by both methods to enhance accuracy and reduce false positives in CNV calling. The patterns of deletions and duplications are shown in the Supplementary Figure 2D and 2E.

2. **Technical Performance Specifications of C-MoKa for Diverse Chromosomal SVs and CNVs**
(1) For balanced translocations, the minimum stably detectable fragment size is 500 kb, with a breakpoint mapping resolution of 100 kb.
(2) Robertsonian translocations and complex balanced translocations are also reliably detectable, sharing the same fragment resolution (500 kb) and breakpoint resolution (100 kb).
(3) For inversions, the required fragment size for reliable detection is 5 Mb, while the breakpoint resolution remains at 100 kb.
(4) Insertions can be detected when the inserted fragment is at least 500 kb in size, with a breakpoint resolution of 100 kb.
(5) For CNVs (deletions and duplications), the minimum stable detectable fragment size is >100 kb (i.e., events larger than 100 kb are confidently called), and the breakpoint resolution is refined to 20 kb.
3. **Additional Practical Information of C-MoKa Compared With SOC Testing in Prenatal Setting**

The current C‑MoKa workflow from sample receipt to final clinical report is as follows: Sample cultured for 7 days, 0.5 day for manual sample processing (aminotic cell fixation by formaldehyde), overnight for automated library construction, 1-2 days paired‑end sequencing on the DNBSEQ‑T7 platform (30 million read‑pairs per sample), and 1-2 days for analysis and report (SV/CNV/LOH calling and interpretation). In total, the entire process can be completed within 10-11 days from sample accession to report delivery. This is substantially shorter than conventional karyotyping (which typically requires 3-4 weeks for cell culture, harvesting, and G‑banding analysis), but a little longer than CMA (4-5 days for DNA extraction, hybridization, scanning, and analysis) and WES (5-6 days for DNA extraction, library construction, sequencing, and analysis) in most diagnostic laboratories due to additional cell culture for C‑MoKa.

Owing to the continuous decline in next‑generation sequencing costs, we have set the total price of the C‑MoKa test at ¥2,500 RMB (approximately US$350) per sample. This pricing is determined by several key factors: (i) the reduced sequencing expenditure achieved through economies of scale and improved reagent efficiency; (ii) the adoption of automated library preparation, which substantially minimizes manual labour and shortens hands‑on time; (iii) the streamlined bioinformatics pipeline that requires limited computational resources and minimal manual intervention; and (iv) the fact that C‑MoKa consolidates multiple assays (karyotyping, CNV detection, and SV analysis) into a single test. Collectively, these factors enable a cost‑effective solution that is competitive with, and often lower than, the combined expense of conventional SOC methods (KT + CMA), while simultaneously providing added clinical value through the detection of balanced SVs and complex rearrangements that would otherwise be missed.

The table below was used to compare the overall costs and turnaround times (TAT) of four prenatal testing technologies in this study: C-MoKa, CMA, iWES, and karyotyping (KT).

| **Parameter** | **C-MoKa** | **CMA** | **iWES** | **KT** |
| --- | --- | --- | --- | --- |
| **Detection platform** | BGI DNBSEQ-T7 platform | CytoScan™ 750K Array | BGI DNBSEQ-T7 platform | AutoVision |
| **Cost** | 2500 RMB | 4800 RMB | 4800 RMB | 378 RMB |
| **Total data** | 30M reads | 550,000‌ CNVs + 200,000‌ SNPs | 45M reads | / |
| **Time-cost (experiment)** | 9-10 days, including 7 days for cell culture, 0.5 day for manual processing; overnight for automated processing; and1-2 days for sequencing. | 4-5 days | 5-6 days | 3-4 weeks, including1-2 weeks for cell culture; One-week experimental operation |
| **Time-cost (analysis)** | 1 day | 1 day | 1 day | one week |
| **Coverage** | Whole-genome | Whole-genome | Whole-exome | At the cellular level |
